# Genomic selection strategies for clonally propagated crops

**DOI:** 10.1007/s00122-023-04300-6

**Published:** 2023-03-23

**Authors:** Christian R. Werner, R. Chris Gaynor, Daniel J. Sargent, Alessandra Lillo, Gregor Gorjanc, John M. Hickey

**Affiliations:** 1 The Roslin Institute and Royal (Dick) School of Veterinary Studies, Easter Bush Research Centre, University of Edinburgh, Midlothian, EH25 9RG UK; 2 NIAB EMR, New Road, East Malling, Kent, ME19 6BJ UK; 3 East Malling Enterprise Centre, Driscoll’s Genetics Ltd, New Road, East Malling, Kent, ME19 6BJ UK

## Abstract

**Key message:**

For genomic selection in clonally propagated crops with diploid (-like) meiotic behavior to be effective, crossing parents should be selected based on genomic predicted cross-performance unless dominance is negligible.

**Abstract:**

For genomic selection (GS) in clonal breeding programs to be effective, parents should be selected based on genomic predicted cross-performance unless dominance is negligible. Genomic prediction of cross-performance enables efficient exploitation of the additive and dominance value simultaneously. Here, we compared different GS strategies for clonally propagated crops with diploid (-like) meiotic behavior, using strawberry as an example. We used stochastic simulation to evaluate six combinations of three breeding programs and two parent selection methods. The three breeding programs included (1) a breeding program that introduced GS in the first clonal stage, and (2) two variations of a two-part breeding program with one and three crossing cycles per year, respectively. The two parent selection methods were (1) parent selection based on genomic estimated breeding values (GEBVs) and (2) parent selection based on genomic predicted cross-performance (GPCP). Selection of parents based on GPCP produced faster genetic gain than selection of parents based on GEBVs because it reduced inbreeding when the dominance degree increased. The two-part breeding programs with one and three crossing cycles per year using GPCP always produced the most genetic gain unless dominance was negligible. We conclude that (1) in clonal breeding programs with GS, parents should be selected based on GPCP, and (2) a two-part breeding program with parent selection based on GPCP to rapidly drive population improvement has great potential to improve breeding clonally propagated crops.

**Supplementary Information:**

The online version contains supplementary material available at 10.1007/s00122-023-04300-6.

## Introduction

In this paper, we show that, for genomic selection (GS) in clonally propagated crops with diploid (-like) meiotic behavior to be effective, crossing parents should be selected based on genomic predicted cross-performance (GPCP), unless dominance is negligible. In most plant and animal breeding programs that apply genomic selection (GS), new parents are selected based on their genomic estimated breeding value (e.g., Meuwissen et al. 2016; Crossa et al. 2017). The genomic estimated breeding value (commonly referred to as GEBV) is the sum of the average effects of an allele predicted for all marker alleles of a genotype. Dominance deviation, which cannot be directly passed on to the progeny, is not considered in the GEBV (Goddard 2009; Su et al. 2012). Selection based on the GEBV aids breeders in increasing the frequency of alleles with beneficial additive genetic effects in a breeding population. As a result, heterozygosity is reduced. Although selection for the GEBV will increase the additive value over time, it may lead to a reduction of the dominance value, unless dominance is negligible. In the long term, using the GEBV to select parents in breeding programs which deliver outbred varieties, such as in clonal plant breeding programs, might not be a suitable method to maximize the total genetic value of the breeding population in a sustainable fashion.

Many major food crops, including nearly all types of fruit and all important roots and tubers, are clonally propagated (Grüneberg et al. 2009; Bradshaw 2016). In clonal breeding programs, new genotypes are created by sexual reproduction and multiplied through clonal propagation (Bisognin 2011; Gemenet and Khan 2017). Breeders use multiple stages of testing to identify the best genotypes in their breeding population. Genotypes are first tested as seedlings in unreplicated trials. Clonal propagation is then used to create genetically identical plants from selected seedlings. As the testing progresses, the number of genotypes is successively reduced and those remaining are tested more intensively across multiple environments and years. The selected genotypes are used to achieve two specific objectives:

i) Generation of an improved offspring population via recombination of selected parents.
ii) Release of the best genotypes as improved clonal varieties.

The time from recombination to variety release spans several years. Traditionally, selection is based on phenotypic performance and the next generation’s parents are selected in the later stages of a breeding program, which leads to long generation intervals (Bradshaw 2016).

Genomic selection offers great potential to optimize the identification of the best clones for variety development, and the selection of crossing parents. Genomic selection exploits associations between genomic markers and phenotypes to predict the value of genotypes based on their genomic marker profiles (Goddard and Hayes 2007). The implementation of GS provides three key advantages:

i) The generation interval can be reduced since parents can be selected as soon as they are genotyped.
ii) The selection accuracy can be increased, especially in early testing stages of a breeding program where the number of locations and replications is low.
iii) The selection intensity can be increased, for example, by genotyping and predicting more genotypes than could be tested in the field.

These advantages allow for several opportunities to reorganize conventional breeding programs. For example, Gaynor et al. (2017) presented an inbred line two-part breeding program employing GS, which reorganized a plant breeding program into:

i) A population improvement component to develop improved germplasm through rapid recurrent GS, and
ii) A product development component to identify genotypes for variety development.

In stochastic simulation, the two-part breeding program doubled the rate of genetic gain relative to a conventional breeding program without increasing cost.

In a clonal breeding program, the reorganization in two parts combined with GS would allow breeders to minimize the generation interval and could substantially increase selection accuracy at the seedling stage.

The generation interval could be reduced to one year or even less since parents can be selected as soon as the seedlings are genotyped. For example, the generation interval in conventional strawberry breeding programs can be four to five years due to the time it takes to generate sufficient phenotypic records to accurately assess a genotype. Genomic selection applied in the seedling stage could result in up to five times the genetic gain achieved in a conventional strawberry breeding program in the same amount of time if the three other components in the breeder’s equation (i.e., selection intensity, diversity, and selection accuracy) remained constant.

The selection accuracy in the seedling stage could be increased since GS enables selection of seedlings based on their predicted performance as clones instead of their phenotypic performance per se. This is achieved when the GS model is trained using clonal phenotypes. In clonal breeding programs, the seedling stage represents a severe genetic bottleneck; in conventional strawberry breeding programs, only a few hundred genotypes among 10,000–20,000 unreplicated seedlings are advanced to the next stage. Selection accuracy is extremely low at the seedling stage for three reasons (Grüneberg et al. 2009), which are:

i) Seedlings and clones derived from those seedlings can differ in their morphology and performance although they are genotypically identical.
ii) Seedlings and clones are often grown in different environments. For example, in European strawberry breeding programs, seedlings are grown in matted rows on the soil and clones are grown as single-pot plants on highly controlled tabletop systems.
iii) Single plant assessment of mostly general appearance and/or a few key traits in the seedling stage shows low heritability and has a low correlation with the breeding goal trait (e.g., yield).

Replacing phenotypic selection in the seedling stage with GS based on clonal phenotypes removes all three obstacles in one step. It also allows for early prediction of traits that are typically not evaluated until later stages of the breeding program, e.g., flavor and shelf life.

In clonally propagated crops, however, dominance may affect the performance of breeding programs which implement GS. The genotypes in clonally propagated crops are typically heterozygous. The genetic value of heterozygous genotypes is a function of additive and non-additive gene action (Falconer and Mackay 1996). If, for the sake of simplicity, epistasis is ignored, the non-additive gene action is entirely defined by dominance. While the differences in the genetic values between genotypes are based on both additive and non-additive genetic effects, it is the additive genetic component which defines long-term genetic gain in a breeding population (Bradshaw 2016). Hence, breeders face the challenging task of having to increase the additive value over time while simultaneously maintaining the dominance value via selection and recombination of parents. The relative importance of these two targets is a function of the dominance degree at the loci affecting the trait under consideration, which is mostly unknown. In strawberry, non-additive effects have been shown to be important for numerous yield component traits, quality traits, and agronomic traits (Shaw et al. 1987; Shaw 1990; Whitaker et al. 2012; Zingaretti et al. 2021), and various experiments have demonstrated substantial reductions in mean performance due to inbreeding depression (Comstock et al. 1958; Niemirowicz-Szczytt 1989; Shaw 1995, 1997; Rho et al. 2012). In cassava, Wolfe et al. (2021) reported significant inbreeding depression for yield using a marker-based directional dominance model. Their results were in accordance with previous studies on inbreeding depression (Pujol and Mckey 2006; Rojas et al. 2009; de Freitas et al. 2016; Kawuki et al. 2011).

We hypothesize that genomic prediction of cross-performance (GPCP) is a better method to select parents in a clonal breeding program than using the GEBV. When GPCP is used, pairs of parents are selected based on the expectation of the total genetic value of their progeny. Genomic prediction of cross-performance could allow breeders to simultaneously increase the frequency of alleles with beneficial additive effects and maintain heterozygosity in the population to exploit dominance effects. In the long term, using GPCP to select parents in a clonal breeding program could be an efficient method to sustainably maximize the total genetic value of the breeding population.

To test our hypothesis, we used stochastic simulation to evaluate three breeding programs and two parent selection methods to deploy GS in clonally propagated crops with diploid (-like) meiotic behavior under different dominance degrees. Strawberry was used as an example.

The three breeding programs included:

i) A breeding program that introduced GS in the first clonal stage, and
ii) Two variations of a two-part breeding program (Gaynor et al. 2017) with one and three crossing cycles per year, respectively.

The two parent selection methods were:

i) Selection of parents based on genomic estimated breeding values (GEBV), and
ii) Selection of parents based on genomic predicted cross-performance (GPCP).

The six combinations of breeding program and parent selection method were compared to a conventional breeding program using phenotypic selection. The structure and key simulation parameters of the conventional breeding program were guided by a commercial strawberry breeding program in the UK.

We observed that the breeding programs using GPCP to select parents produced faster genetic gain than parent selection based on GEBVs unless dominance was negligible. The highest rates of genetic gain were generated by the two-part breeding programs with parent selection based on GPCP.

## Materials and methods

Stochastic simulation was used to evaluate six combinations of three breeding programs and two parent selection methods to deploy GS in clonally propagated crops with diploid (-like) meiotic behavior. We simulated a quantitative trait (such as yield) under four different dominance degrees and evaluated the long-term efficacy of the six combinations of breeding program and parent selection method compared to a conventional breeding program using phenotypic selection.

The material and methods are subdivided into two sections. The first section describes the simulation of the founder genotype population, and the second section describes the simulation of the breeding programs.

The simulation of the founder genotype population comprised:

i) Genome simulation: a heterozygous genome sequence was simulated for a hypothetical diploid and clonally propagated crop species.
ii) Simulation of founder genotypes: the simulated genome sequences were used to generate a base population of 60 founder genotypes.
iii) Simulation of genetic values: a single quantitative trait was simulated for all founder genotypes by summing the biological additive and dominance effects at 20,000 quantitative trait nucleotides. Four different dominance degrees were simulated including 0, 0.1, 0.3 and 0.9.
iv) Simulation of phenotypes: phenotypes were simulated by adding a randomly sampled error to the total genetic value of a genotype.

The simulation of the breeding programs comprised:

i) Recent (burn-in) breeding phase: a conventional phenotypic selection breeding program for clonally propagated crops was simulated for a period of 20 years to provide a common starting point for the future breeding phase.
ii) Future breeding phase: six combinations of three breeding programs and two parent selection methods to deploy GS in clonally propagated crops were simulated and compared to the conventional breeding program for 20 years. In detail, we describe:
  - The GS model.
  - The two parent selection methods including parent selection based on GEBVs and parent selection based on GPCP.
  - The three breeding programs with GS including a breeding program which implemented GS in the first clonal stage, and two variations of a two-part breeding program which implemented GS in the seedling stage with one and three crossing cycles per year, respectively.
  - Comparison of the breeding programs based on parameters measured in the first clonal stage.

### Simulation of the founder genotype population

#### Genome simulation

A heterozygous genome sequence was simulated for each genotype of a hypothetical diploid and clonally propagated crop species. The genome consisted of 20 chromosome pairs with a physical length of 10^8^ base pairs and a genetic length of 100 centimorgans (cM), resulting in a total genetic length of 2000 cM comparable to that of the *Fragaria* × *ananassa* genome (Sargent et al. 2009, 2016; van Dijk et al. 2014; Bassil et al. 2015). The chromosome sequences were generated using the Markovian coalescent simulator (MaCS; Chen et al. 2009), which was deployed using AlphaSimR version 0.11.0 (Gaynor et al. 2019). Recombination rate was derived as the ratio between genetic length (linkage map length) and physical genome length (genome sequence) in base pairs (i.e., 100 cM/10^8^ base pairs = 10^–8^). The per-site mutation rate was set to 2.5 × 10^–8^ mutations per base pair. Effective population size (N_e_) was set to 100 and resulted from a simulated coalescence process with an effective population size of 500, 1250, 1500, 3500, 6000, 12,000 and 100,000 set for 100, 500, 1000, 5000, 10,000, and 100,000 generations ago. Successive reduction of the effective population size was used to reflect a progressive restriction of genetic variance due to natural and artificial selection. The purpose of the coalescence process was to create linkage disequilibrium in the founder population (Fig. S1).

#### Simulation of founder genotypes

The simulated genome sequences were used to generate a base population of 60 diploid founder genotypes in Hardy–Weinberg equilibrium. These genotypes were formed by randomly sampling 20 chromosome pairs per genotype and served as parents in the burn-in phase. A set of 1000 biallelic quantitative trait nucleotides (QTN) and 1000 single nucleotide polymorphisms (SNP) were randomly sampled along each chromosome to simulate a quantitative trait that was controlled by 20,000 QTN and a SNP marker array with 20,000 markers. We chose 20,000 QTN considering the high number of 108,087 protein-coding genes annotated to the octoploid *Fragaria* × *ananassa* reference genome (Edger et al. 2019).

#### Simulation of genetic values

Genetic values for a single quantitative trait, such as yield, were simulated by summing the genetic effects at the 20,000 QTN. Three types of biological QTN effects were modeled to simulate genetic values: additive effects, dominance effects and genotype-by-year (G × Y) interaction effects. Under the AlphaSimR framework, this is referred to as an ADG trait. We will give only a summary of the modeling procedure, while a detailed description can be found in the vignette of the package (Gaynor et al. 2021).

Biological additive effects (*a*) were sampled from a standard normal distribution and scaled to obtain an additive variance of $\sigma_{{\text{A}}}^{2} = 1$ in the founder population. Locus-specific genotype-by-year interaction effects ($g\left( {x, w} \right)$) were modeled using an environmental covariate and a genotype-specific slope:

$$g\left( {x, w} \right) = w*b\left( x \right)$$

The environmental covariate ($w$) represented the random environmental component of the G × Y interaction and was sampled for each year of the simulation from a standard normal distribution. The genotype-specific slope ($b\left( x \right)$) represented the genetic component of the G × Y interaction (sensitivity to changes in the environment), with $x$ being the genotype dosage (number of copies of the alternative allele) at a locus. The effects for the genotype-specific slope were sampled from a standard normal distribution and scaled to obtain a G × Y interaction variance of $\sigma_{{{\text{G}} \times {\text{Y}}}}^{2} = 2\sigma_{{\text{A}}}^{2} = 2$ in the founder population.

Biological dominance effects (*d*) were calculated by multiplying the absolute value of a QTN’s additive effect ($a_{i}$) by a locus-specific dominance degree ($\delta_{i}$). A dominance degree of 0 represents no dominance, and a dominance degree of 1 represents complete dominance. Dominance degrees between 0 and 1 correspond to partial dominance, and values above 1 correspond to overdominance. Dominance degrees were sampled from a normal distribution with mean dominance coefficient $\mu_{\delta }$ and variance $\sigma_{\delta }^{2}$:

$$\delta_{i} { }\sim N\left( {{ }\mu_{\delta } ,\sigma_{\delta }^{2} { }} \right)$$

The dominance effect of QTN $i$ was calculated as:

$$d_{i} = { }\left\{ {\begin{array}{*{20}c} 0 \\ {\delta_{i} *\left| {{ }a_{i} } \right|} \\ \end{array} { }\begin{array}{*{20}c} {\text{if QTN is homozygous}} \\ {\text{ if QTN is heterozygous}} \\ \end{array} } \right.$$

Three levels of average dominance degrees, 0.1, 0.3 and 0.9, were used to simulate overall positive directional dominance and were compared to zero dominance (i.e., additive genetic control only). The variance $\sigma_{\delta }^{2}$ was set to 0.2. Because of the random sampling process, negative dominance degrees at individual loci were possible, resulting in negative dominance interaction effects. The dominance variance ($\sigma_{{\text{D}}}^{2} )$ was then calculated based on the simulated dominance effects. Since no initial covariance between $\sigma_{{\text{A}}}^{2}$ and $\sigma_{{\text{D}}}^{2}$ was simulated, the total genetic variance in the founder population was $\sigma_{{\text{G}}}^{2} = \sigma_{{\text{A}}}^{2} + \sigma_{{\text{D}}}^{2}$.

#### Simulation of phenotypes

Phenotypes were generated by adding random error to the genetic values. The random error was sampled from a normal distribution with mean zero and an error variance $\sigma_{{\text{e}}}^{2}$ defined by the target level of heritability at each testing stage with reference to $\sigma_{{\text{G}}}^{2}$ in the founder population. Entry-mean narrow-sense heritabilities ($h^{2}$) were set to 0.1 in the seedling stage and 0.3 in the first clonal stage. Entry-mean narrow-sense heritabilities in later stages increased due to an increased number of replications per genotype (Table 1). Narrow-sense heritabilities were calculated using the following equation:

$$h^{2} = \frac{{\sigma_{{\text{A}}}^{2} }}{{\sigma_{{\text{P}}}^{2} }} = \frac{{\sigma_{{\text{A}}}^{2} }}{{\sigma_{{\text{A}}}^{2} + \sigma_{{\text{D}}}^{2} + {{\sigma_{{\text{e}}}^{2} } \mathord{\left/ {\vphantom {{\sigma_{{\text{e}}}^{2} } n}} \right. \kern-0pt} n}}}$$

**Table 1:** Number of tested genotypes, replications (Reps), and narrow-sense heritabilities used in the conventional breeding program

Year	Stage	Tested genotypes	Reps	Narrow-sense heritability (*h*^*2*^)*
1	Seedling stage	15,000	1	0.10
2	Clonal stage 1	1000	1	0.30
3	Clonal stage 2	100	2	0.46
4	Clonal stage 3	20	4	0.63
5	Clonal stage 4	5	6	0.72
6	Clonal stage 5	5	6	0.72

*Entry-mean values based on the $\sigma_{{\text{A}}}^{2} :\sigma_{{\text{P}}}^{2}$ ratio in the founder population.

with *n* being the number of replications per genotype and all variance components as defined above.

### Simulation of the breeding programs

#### Recent (burn-in) breeding phase

**Fig. 1:**
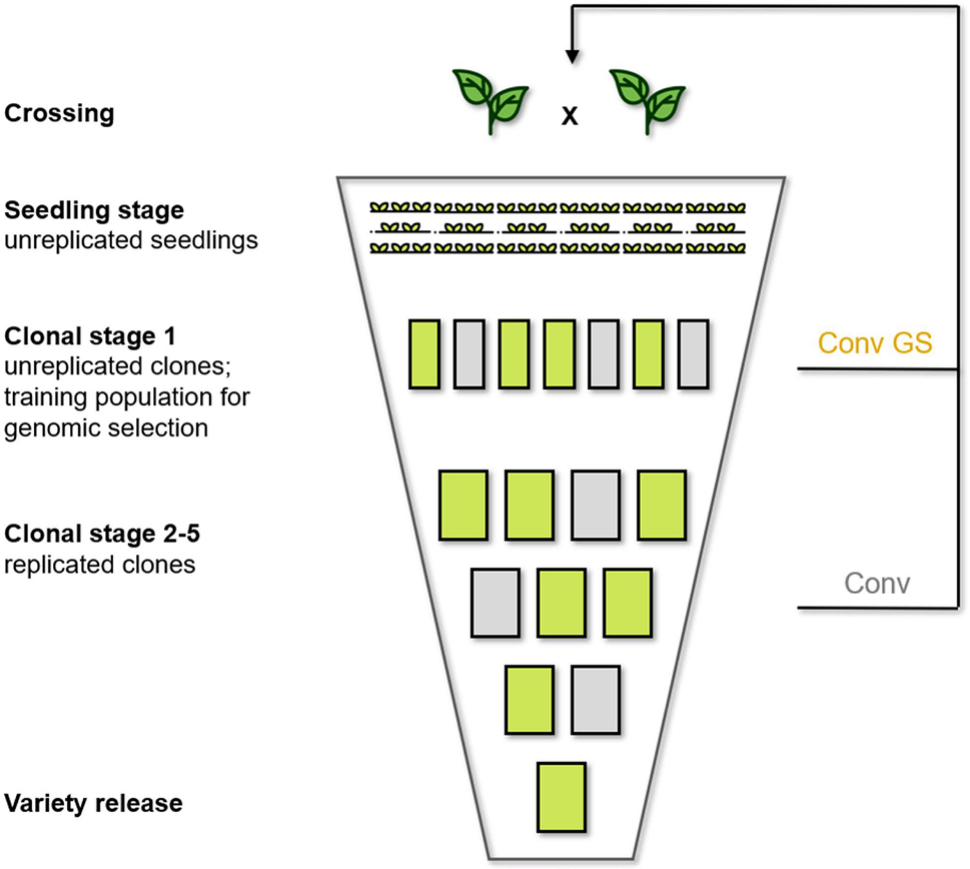
Schematic overview of the conventional breeding program and the conventional breeding program with genomic selection. The conventional breeding program (Conv) was used in the burn-in breeding phase and served as a control in the future breeding phase. In the conventional breeding program, parents were selected in clonal stages 2–5. The conventional breeding program with genomic selection reduced the generation interval to two years by selecting parents in clonal stage 1 based on either genomic estimated breeding values or genomic predicted cross-performance. The genotypes in clonal stage 1 served as training population

A conventional phenotypic selection breeding program for clonally propagated crops was simulated for a period of 20 years (burn-in) to provide a common starting point for the future breeding phase. In combination with the coalescent process, the burn-in phase also served the build-up of linkage disequilibrium in the breeding population (Fig. S1). Each year of the conventional breeding program started with a set of 60 parents planted together in a crossing block. The parents were crossed to generate seedlings, followed by a six-year evaluation period that involved six stages of testing. Selection of parents and selection of the best clones at each stage was based on phenotypes. The structure and key parameter values of the conventional breeding program were guided by a commercial strawberry breeding program in the UK. Figure 1 shows the structure of the conventional breeding program, and Table 1 presents the number of genotypes and replications tested at each stage.

To fill each stage of the simulated breeding pipeline with breeding germplasm prior to the burn-in phase, six cycles of crossing, selection, and advancement of the best genotypes were carried out. Each of these six cycles started with crossing the same 60 founder genotypes to generate 150 F_1_-families with 100 seedlings each, using random sampling of bi-parental crosses without replacement. The best genotypes were then advanced one stage per cycle using phenotypic selection until each stage was filled with a set of genotypes. Replacement of parents was omitted to ensure that total genetic variance in the founder genotypes remained unchanged until the burn-in phase started. The number of founder genotypes was chosen in consultation with strawberry breeders under consideration of the historic origin of their breeding germplasm. Genotype-by-year interaction was ignored during this phase to achieve target-level heritabilities at the beginning of the burn-in phase as defined in Table 1.

In the burn-in phase, selection of parents was carried out in the clonal stages 2, 3, 4 and 5. Each year, the 30 genotypes in the crossing block with the poorest per se performance were replaced by new parents. At first, all 30 genotypes in the clonal stages 3, 4 and 5 were added to the crossing block as new parents if they were not already included. Then, remaining free slots in the crossing block were filled with the best genotypes from the clonal stage 2.

#### Future breeding phase

**Fig. 2:**
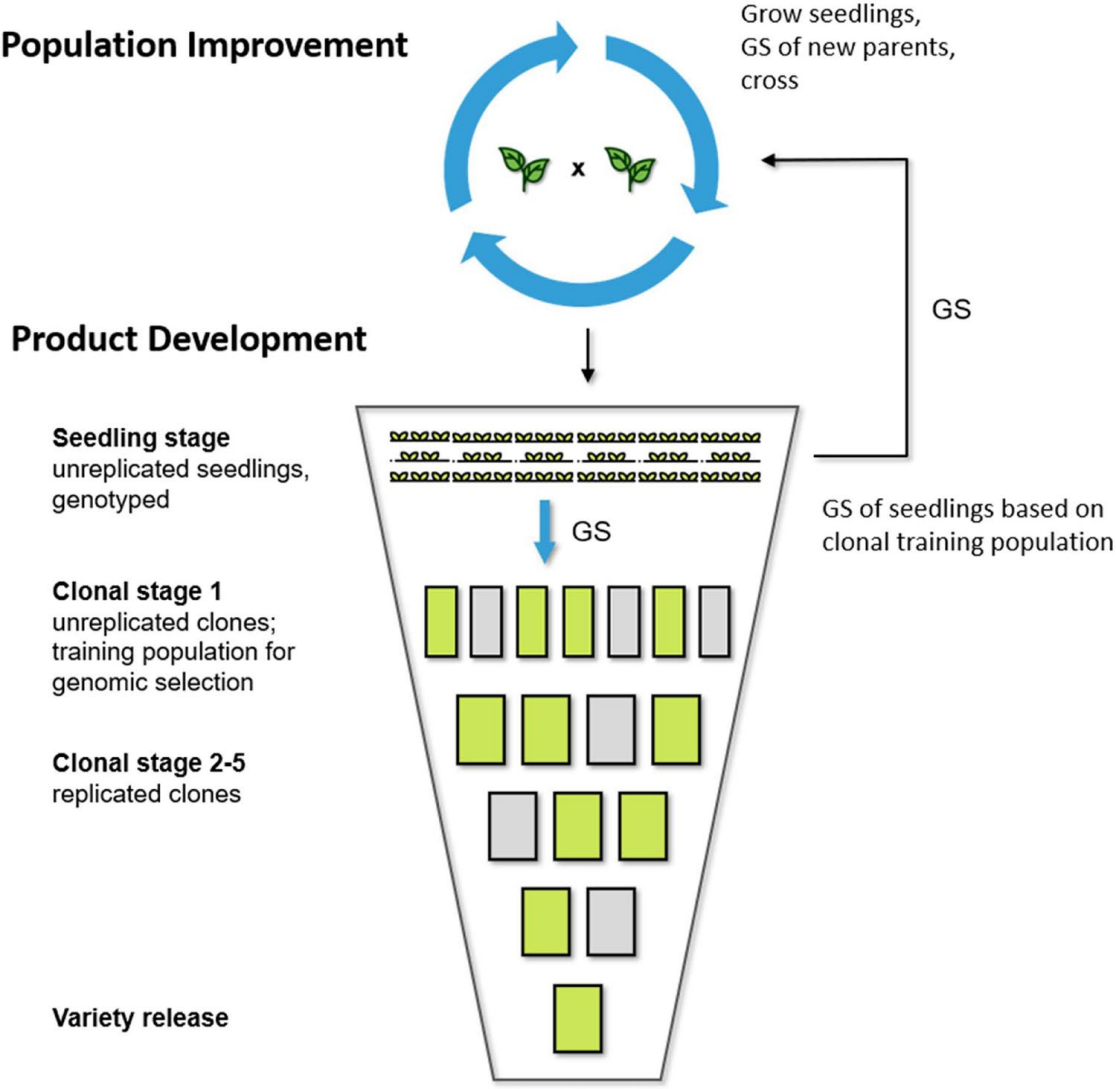
Schematic overview of the two-part breeding program. The two-part breeding program reorganized the conventional breeding program into (1) a population improvement component to develop improved germplasm through rapid recurrent genomic selection; and (2) a product development component to identify the best-performing genotypes. The population improvement component allows to have multiple cycles of crossing and selection per year before the seedlings are advanced to the product development component based on their genomic estimated genetic values. New parents for population improvement were selected based on either genomic estimated breeding values or genomic predicted cross-performance

The future breeding phase was used to evaluate six combinations of three breeding programs and two parent selection methods to deploy GS in clonally propagated crops with diploid (-like) meiotic behavior. These six combinations were simulated for an additional 20 years of breeding and compared to the conventional breeding program. The three GS breeding programs included a conventional breeding program with GS introduced in clonal stage 1 (Fig. 1), and two variations of a two-part breeding program which introduced GS in the seedling stage with one and three crossing cycles per year, respectively (Fig. 2). The two parent selection methods were selection of new parents based on genomic estimated breeding values (GEBVs), and selection of new parents based on genomic predicted cross-performance (GPCP). To obtain approximately equal annual operating costs, the number of seedlings was reduced in the breeding programs with GS to compensate for the additional genotyping costs. Estimated costs were set to $20 for phenotypic evaluation and $25 for array genotyping per genotype after consultation with strawberry breeders. In the two-part breeding programs, all seedlings were genotyped to completely replace phenotypic selection in the seedling stage with GS. Table 2 presents the annual costs for the simulated breeding programs.

**Table 2:** Number of crosses per year, seedlings generated per cross, total number of seedlings planted, number of phenotyped seedlings, number of genotyped individuals, and annual costs of the simulated breeding programs (Conv, conventional breeding program; Conv GS, conventional breeding program with genomic selection; 2Part, two-part breeding program)

Breeding Program	Crosses/year	Seedlings/cross	Seedlings planted	Seedlings phenotyped	Genotyped individuals	Costs ($)
Conv	150	100	15,000	15,000	0	300,000
Conv GS	150	91	13,650	13,650	1000	298,000
2Part (1 cycle)	130	84	11,960	0	11,960	299,000
2Part (3 cycles)	100 × 3	40 × 3	12,000	0	12,000	300,000

To compensate for genotyping costs, the number of seedlings was reduced in the breeding programs with GS

#### Genomic selection model

Genomic predictions were calculated using the following genome-wide regression model presented by Xiang et al. (2016):

$${\varvec{y}} = {\mathbf{X}}{\varvec{\beta}} + {\varvec{f}}b + {\mathbf{Z}}{\varvec{a}} + {\mathbf{W}}{\varvec{d}}^{\user2{*}} + {\varvec{e}}$$

where ${\varvec{y}}$ is a vector of phenotypic entry means, ${\mathbf{X}}{\varvec{\beta}}$ represents years modeled as fixed effects, and ${\varvec{f}}b$ models directional dominance, with ${\varvec{f}}$ modeling genomic inbreeding and *b* being the effect of genomic inbreeding on performance. The genomic inbreeding coefficient of each individual ($f$) was calculated as fraction of homozygous marker loci among all SNP markers, and *b* can be interpreted as genomic inbreeding depression. The vector ${\varvec{a}}$ contains the “biological” additive effects, and ${\varvec{d}}^{\user2{*}}$ is a vector of the dominance effects not captured by ${\varvec{f}}b$. Matrix ${\mathbf{Z}}$ represents the allele dosage (0, 1, 2) of the alternative allele at each marker locus, matrix ${\mathbf{W}}$ is coded 0 for homozygous genotypes and 1 for heterozygous genotypes, and ${\varvec{e}}$ is a vector of residual effects. Random effects *a*, d^*^, and *e* were assumed to be normally distributed with mean zero and variance $\sigma_{{\text{a}}}^{2}$, $\sigma_{{{\text{d}}*}}^{2}$, and $\sigma_{{\text{e}}}^{2}$, respectively.

The effect of *b* was divided by the number of SNP markers and added to ${\varvec{d}}^{\user2{*}}$ to obtain the vector of dominance effects ${\varvec{d}} = {\varvec{d}}^{\user2{*}} + \frac{b}{20,000}$. Additive (*a*) and dominance (*d*) effects were then used to calculate the average effect of an allele for each SNP marker (Varona et al. 2018), and substitution effects were summed to calculate GEBVs. To obtain genomic estimated genetic values (GEGV), the additive and dominance effects were summed. Models were solved in AlphaSimR, using the package’s built-in linear mixed model solver and REML variance component estimation.

The initial training population to train the GS model at the start of the future breeding phase consisted of all the genotypes from clonal stage 1 of the last three years of the burn-in phase. The training population included 3000 genotypes and 3220 phenotypic records. In every year of the future breeding phase, 1000 new genotypes from clonal stage 1 were added to the training population.

#### Parent selection methods

Two parent selection methods were compared for the selection and crossing of parents in the two breeding programs with GS. The first parent selection method will be referred to as parent selection based on *genomic estimated breeding values* (GEBVs). This method represented a conventional “good by good” crossing scheme. The genotypes with the highest GEBVs were selected and used to completely replace the previous year’s crossing block. Crossing was implemented as random sampling of bi-parental combinations without replacement. The second parent selection method will be referred to as parent selection based on *genomic predicted cross-performance* (GPCP). This method implemented systematic selection of bi-parental crosses. The best bi-parental crosses were selected based on the predicted mean genetic values of the F_1_ of a cross. In this way, the average amount of heterosis predicted for the F_1_ due to complementarity between two parents was directly considered in the parent selection process. The mean genetic value of the F_1_ of a cross was predicted based on the equation given by Falconer and Mackay (1996):

$$M_{{{\text{F}}_{1} }} = \mathop \sum \limits_{i = 1}^{n} \left[ {a_{i} \left( {p_{i} - q_{i} - y_{i} } \right) + d_{i} \left[ {2p_{i} q_{i} + y_{i} \left( {p_{i} - q_{i} } \right)} \right]} \right]$$

with $M_{{{\text{F}}_{1} }}$ being the predicted mean genotypic value of the F_1_, $a_{i}$ and $d_{i}$ being the additive and dominance effects of the *n* = 20,000 SNP markers, $p_{i}$ and $q_{i}$ being the frequencies (or dosages) of the two marker alleles measured in one of the two crossing parents, $p_{i}^{\prime }$ and $q_{i}^{\prime }$ being the marker allele frequencies (or dosages) in the second parent, and $y_{i}$ representing the difference in allele frequency (or dosage) between the two parents at the *i*th marker locus, so that $y_{i} = p_{i} - p_{i}^{\prime } = q_{i}^{\prime } - q_{i}$. The concept of the crossing block was abandoned, and no fixed number of parents was selected when GPCP was used.

#### Conventional breeding program with genomic selection

The conventional breeding program with genomic selection introduced GS in clonal stage 1. The structure of the conventional breeding program with genomic selection is shown in Fig. 1. All 1000 genotypes in clonal stage 1 were genotyped to serve as the training population for the GS model. The phenotypic information to train the GS model was obtained from clonal stage 1 to stage 5, so that selected genotypes were represented with up to five separate measurements in the training population due to several years of testing. The model was updated on a yearly basis. When parents were selected based on GEBVs, in each year the best 60 genotypes in clonal stage 1 were used to replace the complete crossing block. When parents were selected based on GPCP, bi-parental cross-performance was predicted for all pairwise cross-combinations in clonal stage 1. The generation interval was two years. Genomic selection was also used to advance the best 100 clones from clonal stage 1 to stage 2 based on their GEGV.

#### Two-part breeding programs

The two-part breeding programs reorganized the conventional breeding program into a population improvement component to develop improved germplasm through rapid recurrent GS, and a product development component to identify genotypes for variety development. Two variations of the two-part breeding program with one and three crossing cycles per year, respectively, were simulated. The structure of the two-part breeding programs is shown in Fig. 2. Genomic selection was introduced in the seedling stage. All seedlings were genotyped and phenotypic selection in the seedling stage was completely replaced by GS. The 1000 genotypes in clonal stage 1 served as training population for the GS model. The phenotypic information to train the GS model was obtained from clonal stage 1 to stage 5, and the model was updated on a yearly basis. Thus, a key feature of the two-part breeding programs is that seedlings were selected using a GS model that was trained with phenotypic records from clones. When parents were selected based on GEBVs, in each crossing cycle, the best 60 seedlings were used to replace the whole crossing block. When parents were selected based on GPCP, bi-parental cross-performance was predicted for all pairwise seedling cross-combinations. The generation interval was one year with one crossing cycle per year, and 1/3 year with 3 crossing cycles per year. Genomic selection was also used to advance the best 1000 seedlings to clonal stage 1 and the best 100 clones from clonal stage 1 to stage 2 based on their GEGV.

#### Comparison of the breeding programs

The performance of the six combinations of three breeding programs and two parent selection methods in comparison with the conventional breeding program was evaluated by measuring the mean total genetic value in clonal stage 1. Each evaluation included ten simulation runs. The mean total genetic value was measured in clonal stage 1 for two reasons:

i) It was the earliest stage in which clones were evaluated.
ii) The general trends observed for genetic gain in clonal stage 1 were representative of genetic gain in the seedling stage and genetic gain in later stages of the breeding programs.

The additive value, the dominance value and genomic inbreeding over time were also measured for the breeding population in clonal stage 1. Genomic inbreeding was measured as the percentage increase in homozygosity at all quantitative trait nucleotides relative to the average homozygosity observed in the founder population. Furthermore, the breeding programs were compared for total genetic variance, additive variance and dominance variance over time, and results are shown in supplementary material (Fig. S8-S10).

Prediction accuracy (Pearson correlation coefficient) was measured in two different ways:

i) In the three breeding programs with GS, prediction accuracy was assessed as the accuracy of the parent selection method (Tab. S2).
ii) In all breeding programs, prediction accuracy was assessed as the prediction accuracy of the total genetic value in the seedling stage (Tab. S3).

## Results

**Fig. 3:**
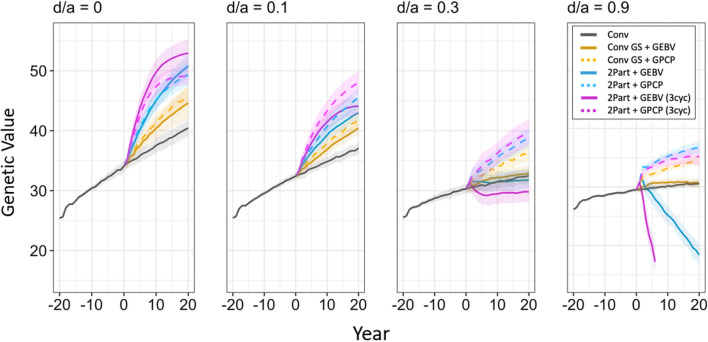
Genetic gain of the simulated breeding programs under different dominance degrees (d/a). In each panel, genetic gain is plotted as the change in the mean genetic value over time in stage 1 for the entire burn-in breeding phase and the future breeding phase. Each line shows the mean genetic value for the 10 simulated replications, and the shading shows the 95% confidence intervals. The different types of breeding program are shown in different colors. The conventional breeding program (Conv) is gray. The conventional breeding program with genomic selection (Conv GS) is yellow. The two-part breeding program with genomic selection (2Part) is shown in blue with one crossing cycle per year and in purple with three crossing cycles per year. The two types of parent selection were shown in different line-styles. Selection based on genomic estimated breeding values (GEBV) is shown by continuous lines. Selection based on genomic prediction of cross-performance (GPCP) is shown by dashed lines

**Fig. 4:**
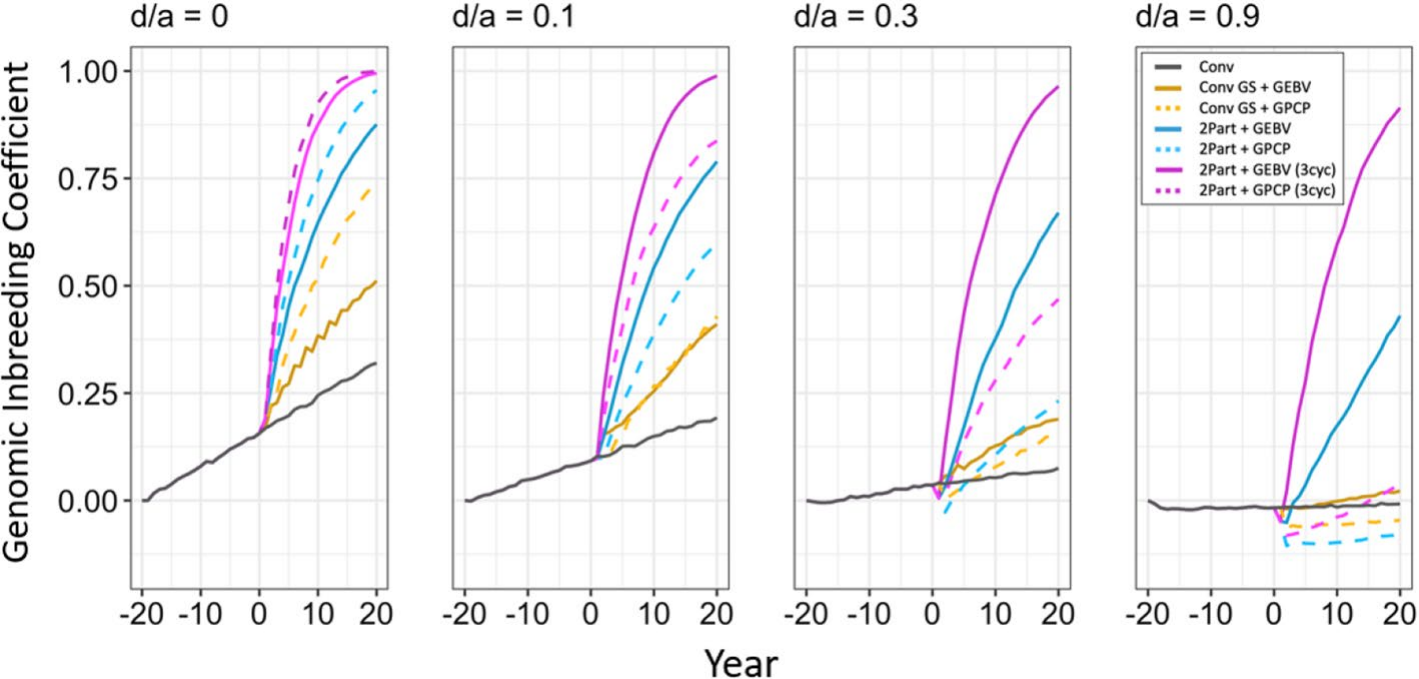
Genomic inbreeding coefficient of the simulated breeding programs under different dominance degrees (d/a). In each panel, the genomic inbreeding coefficient is plotted in stage 1 for the entire burn-in breeding phase and the future breeding phase. Each line shows the mean genomic inbreeding coefficient for the 10 simulated replications. The different types of breeding program are shown in different colors. The conventional breeding program (Conv) is gray. The conventional breeding program with genomic selection (Conv GS) is yellow. The two-part breeding program with genomic selection (2Part) is shown in blue with one crossing cycle per year and in purple with three crossing cycles per year. The two types of parent selection were shown in different line-styles. Selection based on genomic estimated breeding values (GEBV) is shown by continuous lines. Selection based on genomic prediction of cross-performance (GPCP) is shown by dashed lines

**Fig. 5:**
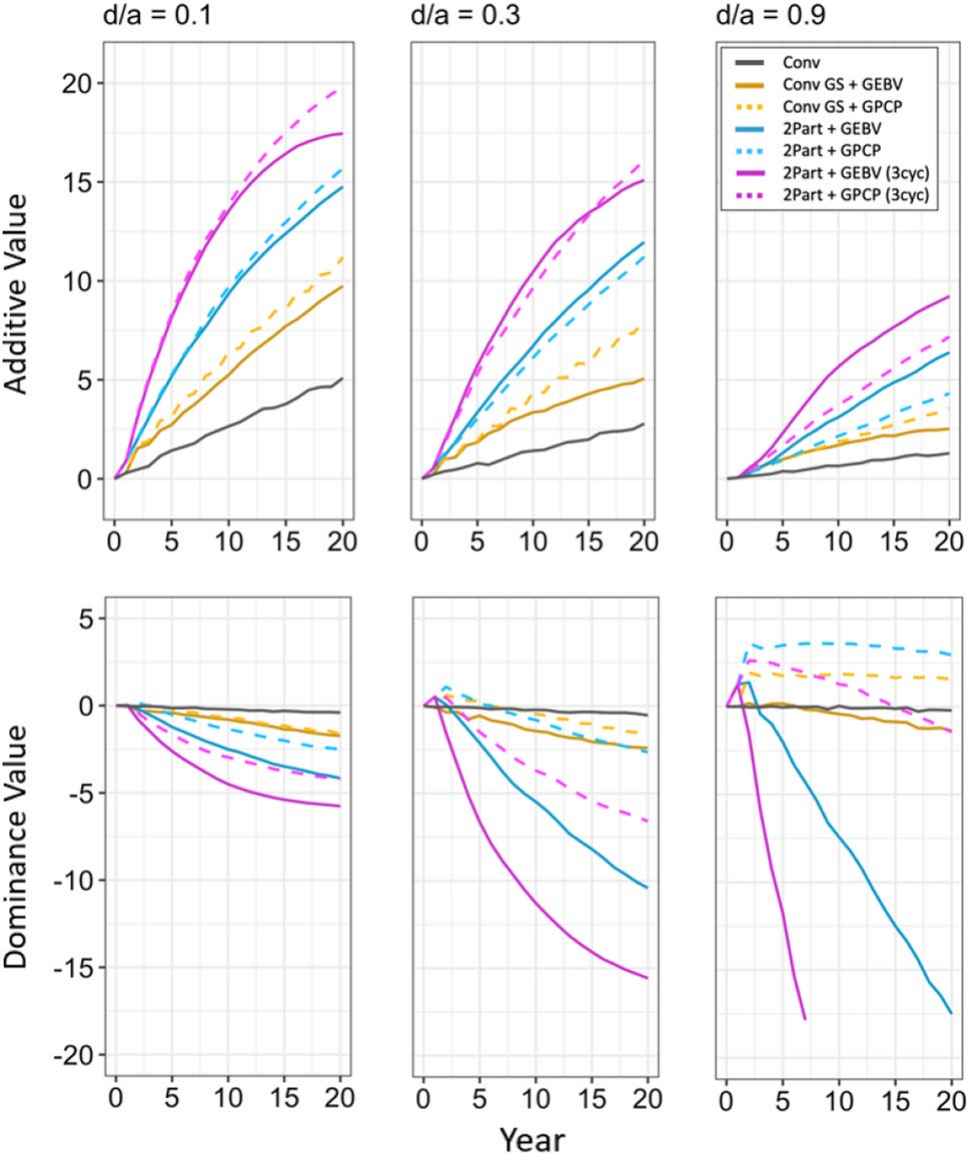
Additive values and the dominance values of the simulated breeding programs under different dominance degrees (d/a). In each of the three top panels, the additive values are plotted in stage 1 for the future breeding phase. The three bottom panels plot the dominance values. Each line shows the mean value for the 10 simulated replications. The different types of breeding program are shown in different colors. The conventional breeding program (Conv) is gray. The conventional breeding program with genomic selection (Conv GS) is yellow. The two-part breeding program with genomic selection (2Part) is shown in blue with one crossing cycle per year and in purple with three crossing cycles per year. The two types of parent selection were shown in different line-styles. Selection based on genomic estimated breeding values (GEBV) is shown by continuous lines. Selection based on genomic prediction of cross-performance (GPCP) is shown by dashed lines. Additive values and dominance values at the beginning of the future breeding phase (year 0) were centered at zero

**Fig. 6:**
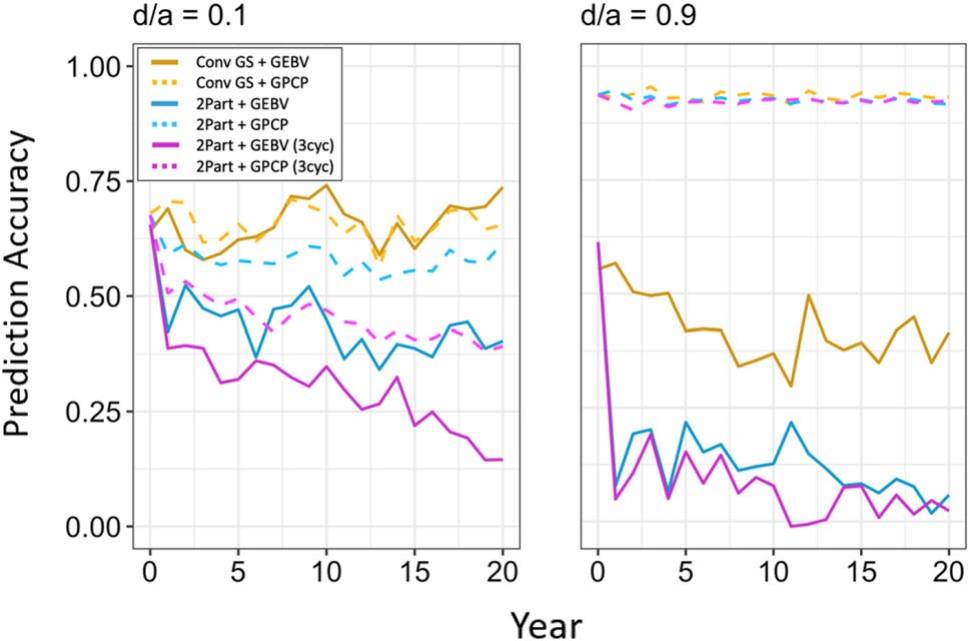
Prediction accuracy for selection of new parents under different dominance degrees (d/a). In each panel, prediction accuracy is plotted for the future breeding phase of the breeding programs with genomic selection. Each line shows the mean prediction accuracy for the 10 simulated replications on an annual basis. The different types of breeding program are shown in different colors. The conventional breeding program with genomic selection (Conv GS) is yellow. The two-part breeding program with genomic selection (2Part) is shown in blue with one crossing cycle per year and in purple with three crossing cycles per year. The two types of parent selection were shown in different line-styles. Selection based on genomic estimated breeding values (GEBV) is shown by continuous lines. Selection based on genomic prediction of cross-performance (GPCP) is shown by dashed lines. Prediction accuracy was measured in the seedling stage for the two-part breeding programs and in stage 1 for the conventional breeding program with genomic selection. Note that the prediction accuracies for all three crossing cycles per year of the two-part breeding program with three crossing cycles are shown in Fig. S11

The results show that for GS in clonally propagated crops with diploid (-like) meiotic behavior to be effective, parents should be selected based on genomic predicted cross-performance (GPCP) unless dominance is negligible. Selection of parents based on GPCP produced faster genetic gain than selection based on GEBVs when the dominance degree was greater than zero (Fig. 3). As the dominance degree increased, selection of parents based on GPCP also produced increasingly more genetic gain than selection based on GEBVs. The two variations of the two-part breeding program with GPCP always produced the most genetic gain unless dominance was negligible. The breeding programs with selection of parents based on GEBVs, on the other hand, produced negative genetic gain when the dominance degree was high. GPCP was advantageous over selection of parents based on GEBVs because it reduced inbreeding in the breeding population when the dominance degree increased (Fig. 4). This enabled better exploitation of the additive value and the dominance value simultaneously, which became more important as the dominance degree increased (Fig. 5). Additionally, GPCP became more accurate, and selection of parents based on GEBVs became less accurate at higher dominance degrees (Fig. 6).

### Genetic gain

Selection of parents based on GPCP produced faster genetic gain than selection based on GEBVs unless dominance was negligible. This is shown in Fig. 3, which plots genetic gain as the mean genetic value against time in clonal stage 1. The four panels show genetic gain under the different simulated dominance degrees for four types of breeding programs and two types of parent selection. As the dominance degree increased, GPCP produced increasingly more genetic gain than selection of parents based on GEBVs.

The three breeding programs with GPCP always produced more genetic gain than the conventional breeding program. The two variations of the two-part breeding program with GPCP always produced the most genetic gain unless dominance was negligible (Fig. 3). When the dominance degree was 0.1, the two-part breeding program gave 2.8 times the genetic gain of the conventional breeding program with one crossing cycle per year, and more than three times the genetic gain with three crossing cycles per year. When the dominance degree was 0.9, it gave almost seven times the genetic gain of the conventional breeding program with one crossing cycle per year, and more than five times the genetic gain with three crossing cycles per year.

Figure 3 also shows that the two-part breeding program with parent selection based on GEBVs and three crossing cycles per year generated the most genetic gain when the dominance degree was zero. However, after a sharp increase in the first few years, the rate of genetic gain drastically decreased and started to approach a plateau. The two-part breeding program with parent selection based on GEBVs and one crossing cycle per year generated the second most genetic gain. In the first few years, it showed a lower rate of genetic gain than the two-part breeding programs with GPCP. In the long term, however, both two-part breeding programs with GPCP started to plateau and were outperformed by the two-part breeding program with parent selection based on GEBVs and one crossing cycle per year.

Figure 3 also shows that selection of parents using GEBVs produced negative genetic gain over time when the dominance degree was high. All breeding programs showed a reduced rate of genetic gain when the dominance degree increased. However, this reduction was stronger when parents were selected using GEBVs. The two-part breeding programs with parent selection based on GEBVs produced even less genetic gain than the conventional breeding program when the dominance degree was 0.3 and 0.9. These results were not surprising as selection of parents based on GEBVs gave a faster increase in the inbreeding coefficient than selection based on GPCP when the dominance degree was high, which resulted in inbreeding depression.

### Genomic inbreeding coefficient

Selection of parents based on GPCP reduced inbreeding when the dominance degree increased. This is shown in Fig. 4, which plots the genomic inbreeding coefficient against time in clonal stage 1 under the four simulated dominance degrees. As the dominance degree increased, all breeding programs showed a decreased growth rate of the genomic inbreeding coefficient. However, this decrease was much stronger when parents were selected based on GPCP compared to when GEBVs were used.

Figure 4 also shows that the two-part breeding programs with GPCP gave the strongest reduction in the genomic inbreeding coefficient as the dominance degree increased. When the dominance degree was zero, both breeding programs had almost approached complete inbreeding at the end of the future breeding phase. However, when the dominance degree was 0.9, the two-part breeding program with GPCP and one crossing cycle per year gave the lowest inbreeding coefficient, which was negative during the entire future breeding phase. The two-part breeding program with GPCP and three crossing cycles per year was also negative in the first half of the future breeding phase but became positive during the second half.

### Additive values and dominance values

Selection of parents based on GPCP enabled better simultaneous exploitation of the additive and dominance values than selection of parents based on GEBVs. This is shown in Fig. 5, which plots the additive values and the dominance values against time in clonal stage 1. The three top panels show the additive values, and the three bottom panels show the dominance values.

The two-part breeding program with GPCP and three crossing cycles per year gave the highest increase of the additive value over time when the dominance degree was 0.1 and 0.3 (Fig. 5 top). However, when the dominance degree was 0.9, the two-part breeding program with parent selection based on GEBVs and three crossing cycles per year gave the highest increase of the additive value.

Figure 5 (top) also shows that the rate of increase of the additive value over time was reduced in all breeding programs as the dominance degree increased. The conventional breeding program always gave the lowest increase of the additive value.

Selection of parents using GPCP generated increased dominance values as the dominance degree increased (Fig. 5 bottom). It gave a reduction of the dominance value when the dominance degree was 0.1, but a strong increase when the dominance degree was 0.9. The increase of the dominance value compensated for the reduction of the additive value as the dominance degree increased. The two-part breeding programs with GPCP showed the strongest increase. With three crossing cycles per year, however, a rapid decrease of the dominance value over time was observed.

Selection of parents based on GEBVs did not effectively exploit the dominance value as the dominance degree increased. This is also shown in Fig. 5 (bottom). Both variations of the two-part breeding program with parent selection based on GEBVs generated reduced dominance values as the dominance degree increased. This reduction in the dominance value over time became more extreme as the dominance degree increased and exceeded the increase in the additive value when the dominance degree was high.

### Prediction accuracy of the parent selection method

The advantage of GPCP to select parents over using GEBVs was not only due to better simultaneous exploitation of the additive and dominance value, but also resulted from a higher prediction accuracy when the dominance degree was high. At higher dominance degrees, GPCP became more accurate, and selection of parents based on GEBVs became less accurate. This is shown in Fig. 6, which plots the prediction accuracy of the parent selection methods against time under the dominance degrees of 0.1 and 0.9. Prediction accuracy was measured in the seedling stage for the two-part breeding programs and in clonal stage 1 for the conventional breeding program with genomic selection. Prediction accuracy of GPCP became similar in the three GS breeding programs as the dominance degree increased. It should be noted, however, that prediction accuracies might not be directly comparable between breeding programs since the programs showed different trends for their genetic variances at the respective stage of parent selection.

### Prediction accuracy of the genetic value in the seedling stage

**Fig. 7:**
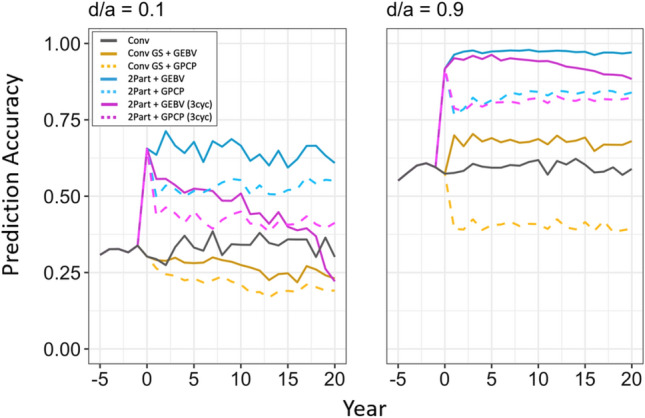
Prediction accuracy for the total genetic value of the seedlings under different dominance degrees (d/a). In each panel, prediction accuracy is plotted in the seedling stage for the last five years of the burn-in breeding phase and the future breeding phase. Each line shows the mean prediction accuracy for the 10 simulated replications. The different types of breeding program are shown in different colors. The conventional breeding program (Conv) is gray. The conventional breeding program with genomic selection (Conv GS) is yellow. The two-part breeding program with genomic selection (2Part) is shown in blue with one crossing cycle per year and in purple with three crossing cycles per year. The two types of parent selection were shown in different line-styles. Selection based on genomic estimated breeding values (GEBV) is shown by continuous lines. Selection based on genomic prediction of cross-performance (GPCP) is shown by dashed lines

Prediction accuracy of the genetic value of the seedlings increased when the dominance degree increased. Figure 7 plots the prediction accuracy of the genetic value in the seedling stage over time under the dominance degrees of 0.1 and 0.9. The highest prediction accuracy was observed in the two-part breeding program with parent selection based on GEBVs and one crossing cycle per year. In all breeding programs, prediction accuracy was lower when parents were selected based on GPCP compared to GEBVs. The conventional breeding program with genomic selection using GPCP to select parents showed the lowest prediction accuracies under all dominance degrees. As for the prediction accuracy of the parent selection method, prediction accuracies might not be directly comparable between breeding programs since the programs showed different trends for their genetic variances in the seedling stage.

## Discussion

For genomic selection in clonally propagated crops with diploid (-like) meiotic behavior to be effective, parents should be selected based on genomic predicted cross-performance (GPCP) unless dominance is negligible. To discuss this result, we first describe how genomic selection of parents can improve clonal breeding programs under the assumption of additive genetic control. We then explain why genomic selection of parents requires consideration of dominance effects when dominance is appreciable. We show that selection of parents based on GPCP enables simultaneous exploitation of additive and dominance effects, which facilitates exploitation of pseudo-overdominance in the progeny of a cross to increase genetic gain when the dominance degree is high. We also show that, at higher dominance degrees, heterozygosity becomes a reliable predictor of the dominance value when parents are selected based on GPCP.

### Genomic selection of parents improved genetic gain under additive genetic control

Under additive genetic control, genomic selection of parents always produced faster genetic gain than phenotypic selection. This was observed regardless of whether parents were selected based on GEBVs or based on GPCP.

As expected, GS improved the conversion of genetic variance into genetic gain. This improvement resulted from a shortened generation interval and an increased selection accuracy in early stages of the breeding program. As new genotypes were added to the training population each year, more information became available to predict the marker effects, while the impact of G × Y interaction on the marker effects was reduced. Therefore, the breeding programs with GS also showed an accelerated depletion of genetic variance over time compared to the conventional breeding program (Fig. S8). This depletion was most severe with three crossing cycles per year.

Our findings under additive genetic control were consistent with those of Gaynor et al. (2017) who used stochastic simulations to evaluate GS strategies in plant breeding programs for developing inbred lines. We refer the reader to this study for a detailed description of the relationship between the generation interval, prediction accuracy, G × Y interaction and genetic variance when additive genetic control is assumed.

### The two-part breeding programs better exploited genomic selection than the conventional breeding program with genomic selection under additive genetic control

The two-part breeding programs showed the highest rates of genetic gain under additive genetic control. The better performance compared to the conventional breeding program with genomic selection resulted from an optimal exploitation of GS with a very short generation interval and an improved selection accuracy in the seedling stage.

Selection in the seedling stage poses a major challenge in conventional clonal breeding programs due to a high selection intensity combined with low selection accuracy (Grüneberg et al. 2009; Bradshaw 2016). The two-part breeding programs improved selection accuracy by replacing phenotypic selection with GS. When phenotypic selection was used, seedlings were selected based on their observed per se performance. When GS was used, seedlings were selected based on their predicted performance as clones.

Genomic selection in the seedling stage increased selection accuracy for two reasons:

i) The phenotypic records in the clonal stages which were used to train the GS model had a higher heritability than the phenotypic records of the unreplicated seedlings.
ii) Marker alleles were replicated within and across multiple years.

This increase in selection accuracy also laid the foundation for the selection of parents in the seedling stage, allowing for one or multiple crossing cycles per year to minimize the generation interval.

In the conventional breeding program with genomic selection applied in clonal stage 1, selection in the seedling stage was based on phenotypic per se performance. Hence, selection accuracy in the seedlings did not increase compared to the conventional breeding program without genomic selection. The increased rate of genetic gain mainly resulted from a shortened generation interval and an increased selection accuracy in clonal stage 1.

### Selection of parents based on genomic predicted cross-performance increased selection intensity compared to selection of parents based on genomic estimated breeding values under additive genetic control

Under additive genetic control, differences in genetic gain between the two parent selection methods likely resulted from an increased selection intensity when parents were selected based on GPCP compared to selection of parents based on GEBVs.

When GEBVs were used, the 60 best genotypes were selected and randomly crossed to mimic a “good by good” crossing scheme. When GPCP was used, bi-parental crosses were selected based on the predicted mean genetic value of the F_1_. Under additive genetic control, the predicted mean genetic value of the F_1_ is equal to the mean GEBV of both parents. Selection of parents based on GPCP resulted in an excessive use of a few very good parents in many crosses, and the number of parents was often less than 60 (Fig. S4). Therefore, the selection intensity was higher compared to when parents were selected based on GEBVs and randomly crossed.

In the conventional breeding program with genomic selection, the increased selection intensity resulted in more genetic gain over time compared to when parents were selected based on GEBVs. In the two-part breeding programs, however, it resulted in more genetic gain in the first years, but thereafter genetic gain reached a plateau due to a depletion of genetic variance. This depletion of genetic variance was more severe when three crossing cycles per year were used.

A crossing strategy in a real-world breeding program would probably lie somewhere in between the two simulated parent selection methods. A breeder would not randomly select crosses, but rather combine parents that are expected to generate improved progeny. Although very good genotypes may be used at high frequency, a breeder would make sure that an overly excessive use is avoided.

### Genomic selection of crossing parents requires consideration of dominance effects unless dominance is negligible

If dominance is appreciable, genetic gain becomes a function of additive and non-additive gene action. If epistasis is ignored, non-additive gene action is completely determined by dominance. Achieving high rates of genetic gain then depends on an efficiently balanced exploitation of additive and dominance effects (Bradshaw 2016).

This requires two opposed actions:

i) The frequency of alleles with beneficial additive genetic effects in homozygous state needs to be increased to improve the additive value of the breeding population.
ii) Heterozygosity needs to be preserved to exploit dominance effects and keep the dominance value high in the breeding population.

A well-balanced exploitation of the additive value and the dominance value can only be accomplished through selection and recombination of suitable parents. While inbreeding can be used to increase the frequency of beneficial alleles in homozygous state to improve the additive value, it also results in a reduction of heterozygosity and the dominance value. As the dominance degree increases, the importance of the dominance value relative to the additive value increases and maintaining or even increasing heterozygosity becomes critical. In the worst-case scenario, a decrease in the dominance value over time would exceed the increase in the additive value, and the rate of genetic gain becomes negative due to inbreeding depression. To ensure high and sustainable rates of genetic gain in clonal breeding programs, a parent selection method is required that optimally balances the contribution of the additive and dominance components in the next generation.

### Selection of parents based on genomic predicted cross-performance enabled simultaneous exploitation of additive effects and dominance effects

Selection of parents based on GPCP enabled efficient simultaneous exploitation of additive effects and dominance effects by reducing the increase in inbreeding over time when the dominance degree increased. This became critical to make positive genetic gain over time when the dominance degree was high.

As the dominance degree increased, selection of parents based on GPCP produced increasingly more genetic gain than selection based on GEBVs. The GEBV is the sum of the average effects of the marker alleles called in a genotype. These average effects are predicted for all markers simultaneously by performing a linear regression of the phenotypes in the training population on the marker genotypes, the concept described by Falconer (1985) for a one-locus model. Although the genomic estimated breeding value thereby generally captures a large part of the dominance interaction (Falconer and Mackay 1996; Hill et al. 2008), this population-based predictor of the value of an individual parent for the progeny generation ignores dominance deviation.

In contrast, GPCP fully captures additive and dominance marker effects and thereby enables prediction of the expected total genetic value of the progeny of a bi-parental cross rather than prediction of the value of an individual parent for the progeny population. The inclusion of non-additive effects can also facilitate an enhancement and an improved exploitation of non-additive genetic variation compared to parent selection based on genomic estimated breeding values (Varona et al. 2018). When parents were selected based on GPCP, the enhancement of non-additive genetic variation was a direct outcome of the reduced increase in inbreeding over time. The improved exploitation of non-additive genetic variation resulted from the efficiently balanced exploitation of the additive and dominance value.

Interestingly, the prediction model used for GPCP autonomously assigned more weight to the predicted dominance value of a cross as dominance increased. This was accomplished by including the genomic inbreeding coefficient (*f*) as a covariate in the model, which accounted for directional dominance and can be seen as an estimator for inbreeding depression explained by genomic inbreeding (Xiang et al. 2016; Varona et al. 2018). As the dominance degree increased, the value of crosses which maintained or even increased heterozygosity in the population also increased. The level of heterozygosity in the progeny population was controlled by the number of parents used for crossing, which increased to minimize or avoid inbreeding when the dominance degree increased (Fig. S4-S7).

### Selection of parents based on genomic predicted cross-performance enabled exploitation of pseudo-overdominance in the progeny of a cross when the dominance degree was high

The two-part breeding programs with parent selection based on GEBVs gave negative genetic gain due to severe inbreeding depression when the dominance degree was high. After the first year, the decrease in the dominance value over time was consistently higher than the increase in the additive value.

At first sight, this might seem surprising as we did not simulate overdominance at the allele-level. Under the one-locus model with a dominance degree < 1, the allele combination with the favorable allele in homozygous state will result in the highest genetic value of all pairwise allele combinations. In this case, selection of parents based on the GEBV is an efficient strategy to increase the frequency of the beneficial allele in the population over time, and hence to increase genetic gain. Only under overdominance does the heterozygote become superior to both homozygotes and the fixation of the favorable allele results in a reduction of the genetic value (Falconer and Mackay 1996).

Overdominance seems to be an extremely rare phenomenon in nature. However, due to linkage disequilibrium (LD), haplotype blocks are the units of genetic transmission rather than single loci. When haplotype blocks with favorable alleles in repulsion phase are combined during sexual recombination, the cumulative effect of these loci can create pseudo-overdominance although the dominance degree at each locus is < 1 (Bingham et al. 1994; Bingham 1998).

Selection of parents based on the GEBV will increase the frequency of the haplotype blocks with the highest sum of average effects. The heterotic effects due to pseudo-overdominance, however, are reduced from one generation to the next. Furthermore, even haplotype blocks with low GEBVs may contain favorable alleles, which are removed from the population through selection. As a result, genetic variance is reduced, limiting long-term additive genetic gain.

Selection of parents based on GPCP, on the other hand, considers the heterotic potential of a cross when predicting the performance of the progeny. In this way, non-additive effects due to complementation of haplotype blocks can be preserved in the population over several generations if their contribution to the total genetic value is high. Furthermore, by preserving haplotype blocks with lower GEBVs for a few generations, recombination can make the favorable alleles that they contain available.

### Multiple crossing cycles per year using genomic prediction of cross-performance without updating the prediction model can adversely affect long-term genetic gain especially when the dominance degree is high

In the two-part breeding programs with parent selection based on GPCP, genomic inbreeding increased faster with three crossing cycles per year compared to one crossing cycle per year. While using three crossing cycles per year resulted in more genetic gain when the dominance degree was low, it gave less genetic gain when the dominance degree was high.

As the dominance degree increased, keeping inbreeding low became critical to ensure a sustainable exploitation of dominance effects. We hypothesize that two factors caused the two-part breeding program with three crossing cycles per year to be less efficient at keeping inbreeding low than the two-part breeding program with one crossing cycle per year:

i) A reduced number of seedlings generated per crossing cycle.
ii) An irregular updating of the prediction model for selection of parents.

The increased number of crossing cycles per year in combination with a reduced number of crosses and seedlings per cross resulted in an accelerated removal of haplotype block diversity from the breeding population. To equalize annual costs, the size of the seedling population was reduced from 12,000 to 4,000 seedlings per cross with three crossing cycles per year. Hence, the population became more susceptible to genetic drift and dominance effects due to complementation of haplotype blocks could not be maintained over multiple generations.

The irregular updating of the prediction model for the selection of parents resulted in a less efficiently balanced exploitation of additive and dominance effects. Although multiple cycles of crossing and selection per year effectively reduced the generation interval, the prediction model was updated only once a year, and cross-prediction became increasingly less efficient. Assuming purely additive gene action in a simulation of a line breeding program, Gaynor et al. (2017) found that the increased genetic distance between the training and prediction population caused selection accuracy to drop with every additional crossing cycle. Although we also observed a reduction in prediction accuracy with an increased number of cycles (Fig. S11), the constant weights assigned to additive and dominance effects by the prediction model contributed more strongly to the accelerated reduction of heterozygosity. While inbreeding increased with every crossing cycle, the covariate associated with genomic inbreeding in the prediction model remained unchanged for two more cycles and could not sufficiently counteract inbreeding.

These results indicate that GPCP might not be optimal to select parents when multiple cycles of crossing and selection are done without updating the prediction model. To solve this problem, a strategy such as optimal contribution selection could be useful to maximize long-term genetic gain as shown by Gorjanc et al. (2017) in a two-part line breeding program with multiple crossing cycles per year.

### Heterozygosity became a reliable predictor of the dominance value when the dominance degree was high

Prediction accuracy of GPCP increased as the dominance degree increased. Furthermore, prediction accuracy of the genetic value of the seedlings increased as the dominance degree increased.

We infer that marker-based heterozygosity became an accurate predictor of non-additive genetic effects for selection of crosses especially when the dominance degree was high, i.e., when the genetic value was mainly a result of non-additive gene action. This was mostly driven by including the genomic inbreeding coefficient (*f*) as a covariate in the model. Selection accuracy in the seedlings also was significantly increased under high dominance degrees. Both factors contributed to the two-part breeding programs with GPCP generating the most genetic gain over time when dominance was appreciable.

### Further implications for breeding programs for outbred species

In this paper, we proposed genomic prediction of cross-performance (GPCP) as an efficient method to select parents in clonal breeding programs based on the predicted mean genetic value of a cross. We expect that GPCP could also be used in other breeding programs for outbred individuals, such as animal breeding programs, to increase rates of genetic gain. As with clonal crops, animal breeding programs must account for the detrimental effects of inbreeding depression. Animal breeders use various methods to accomplish this, ranging from rule-of-thumb recommendations to avoid matings between close relatives to optimal contribution selection, a numeric technique for limiting population-level inbreeding (Woolliams et al. 2015). We hypothesize GPCP to outperform these methods by directly estimating progeny performance and thereby accounting for inbreeding depression in a purely data-driven manner, given the prediction model is constantly updated. Further research, however, will be required to test this hypothesis.

While we showed that GPCP is advantageous over selection of parents based on the GEBV unless dominance is negligible, opportunities to further improve cross-prediction may exist. Wolfe et al. (2021) proposed to combine the predicted total genetic mean and variance of a cross into a usefulness criterion. They predicted the total genetic variance of each cross based on the parents’ marker haplotypes, marker effects, and recombination frequencies. Selection of parents based on the usefulness of a cross might enable even higher rates of genetic gain than selection based on the predicted total genetic mean. A long-term comparison of both methods, however, will be critical to investigate this hypothesis. It should be mentioned that Wolfe et al. selected parents based on GEBVs, a method that we advise against in this paper.

Although we assume that our overall conclusions are generally valid in outbred species with diploid (-like) meiotic behavior, we strongly recommend performing a breeding program-specific analysis when considering practical implementation of GPCP. As with genomic selection strategies in general, the efficiency of GPCP is very likely to be influenced by factors such as training population size, marker density, genotyping costs, and trait heritability. A sensitivity analysis covering all those factors would go far beyond the scope of this study, and its importance can only be emphasized here.

## Supplementary Information

Below is the link to the electronic supplementary material.

Supplementary file1 (PDF 250 KB)

Supplementary file2 (PDF 314 KB)

Supplementary file3 (PDF 540 KB)

Supplementary file4 (PDF 107 KB)

Supplementary file5 (PDF 10 KB)

Supplementary file6 (PDF 29 KB)

## Data Availability

Not applicable.

## Code availability

All simulations were done using AlphaSimR version 0.11.0 (Gaynor et al. 2019). The entire code used to simulate the different breeding programs in AlphaSimR is available at FigShare.
