# Supplementary material for: Genomic selection strategies for clonally propagated crops
Source: Theor Appl Genet. 2023 Mar 23;136(4):74. doi: 10.1007/s00122-023-04300-6 (PMC10036424; doi:10.1007/s00122-023-04300-6)
Supplement: Supplementary file 1 — Supplementary file1 (PDF 250 KB) [file 122_2023_4300_MOESM1_ESM.pdf]

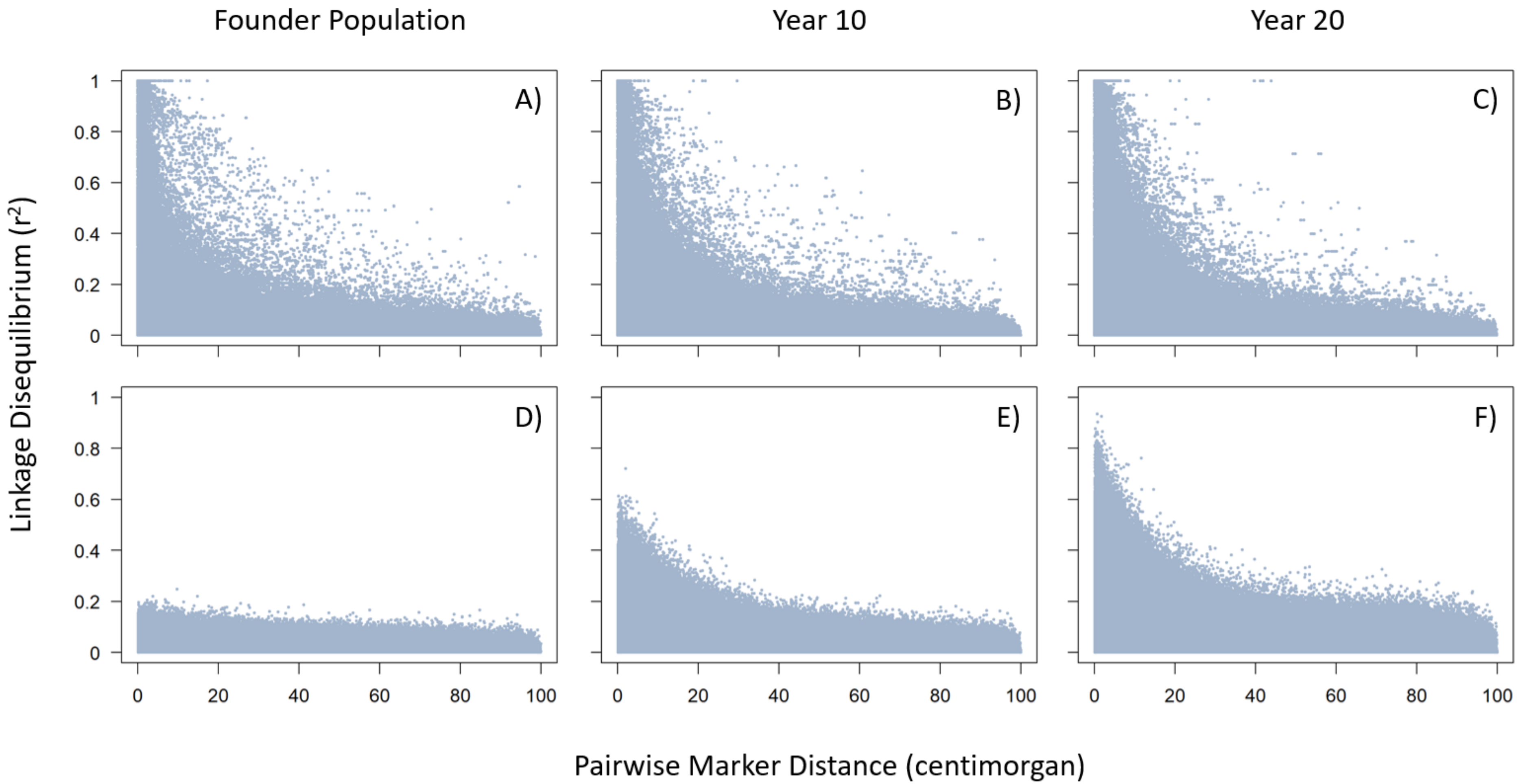

Founder Population
Year 10
Year 20
A)
B)
C)
D)
E)
F)
1
0.8
0.6
0.4
0.2
0
0
20
40
60
80
100
Linkage Disequilibrium (r^2)
Pairwise Marker Distance (centimorgan)

Figure S1 Intra-chromosomal linkage disequilibrium (LD) decay plot as a function of genetic distance in centimorgan (cM) during the burn-in breeding phase. LD estimates between intra-chromosomal marker pairs are plotted for two scenarios at three time points during the burn-in breeding phase to demonstrate the build-up of linkage disequilibrium. The two scenarios include a burn-in breeding phase started from i) a founder population generated with MaCS to create initial LD (A-C), and ii) a founder population in linkage equilibrium (LE) used as a control scenario (D-F). The three time points include the founder population, year 10, and year 20 of the burn-in phase. In both scenarios, we observe a build-up of LD as a result of selection and crossing of parents. At all three time points, intra-chromosomal LD is higher in the burn-in breeding program that started from a founder population generated with MaCS. However, even in the burn-in breeding program that started from a founder population in LE, a build-up of LD can be observed. LD between marker loci and the causal genes is a basis for genomic selection. LD was reported as squared correlation coefficient between pairs of loci (r^2) in stage 1 at a dominance degree of 0.3. Only one replication was run to demonstrate the build-up of LD. Similar patterns were observed at a dominance degree of 0, 0.1, and 0.9. The function "quickHaplo" of the R package AlphaSimR was used to generate a founder population in LE.
