# Supplementary material for: Genomic selection strategies for clonally propagated crops
Source: Theor Appl Genet. 2023 Mar 23;136(4):74. doi: 10.1007/s00122-023-04300-6 (PMC10036424; doi:10.1007/s00122-023-04300-6)
Supplement: Supplementary file 2 — Supplementary file2 (PDF 314 KB) [file 122_2023_4300_MOESM2_ESM.pdf]

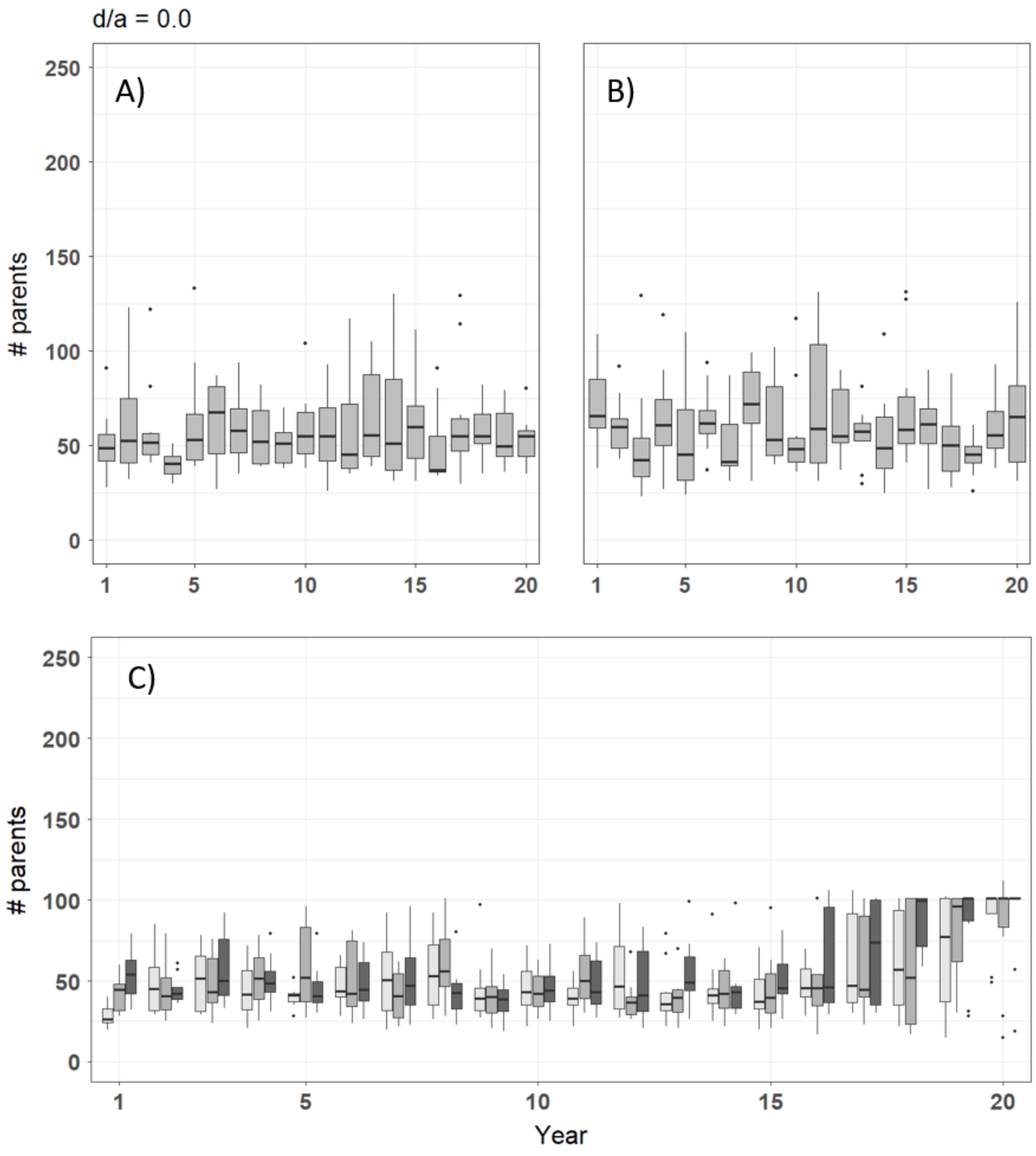

Figure S4 Number of parents used in the breeding programs with parent selection based on genomic predicted cross performance (GPCP) under additive genetic control (d/a = 0). The number of parents used in the 10 simulation replications is shown as boxplots for the conventional breeding program with genomic selection (A), the two-part breeding program with one crossing cycle per year (B), and the two-part breeding program with three crossing cycles per year (C) during the future breeding phase.

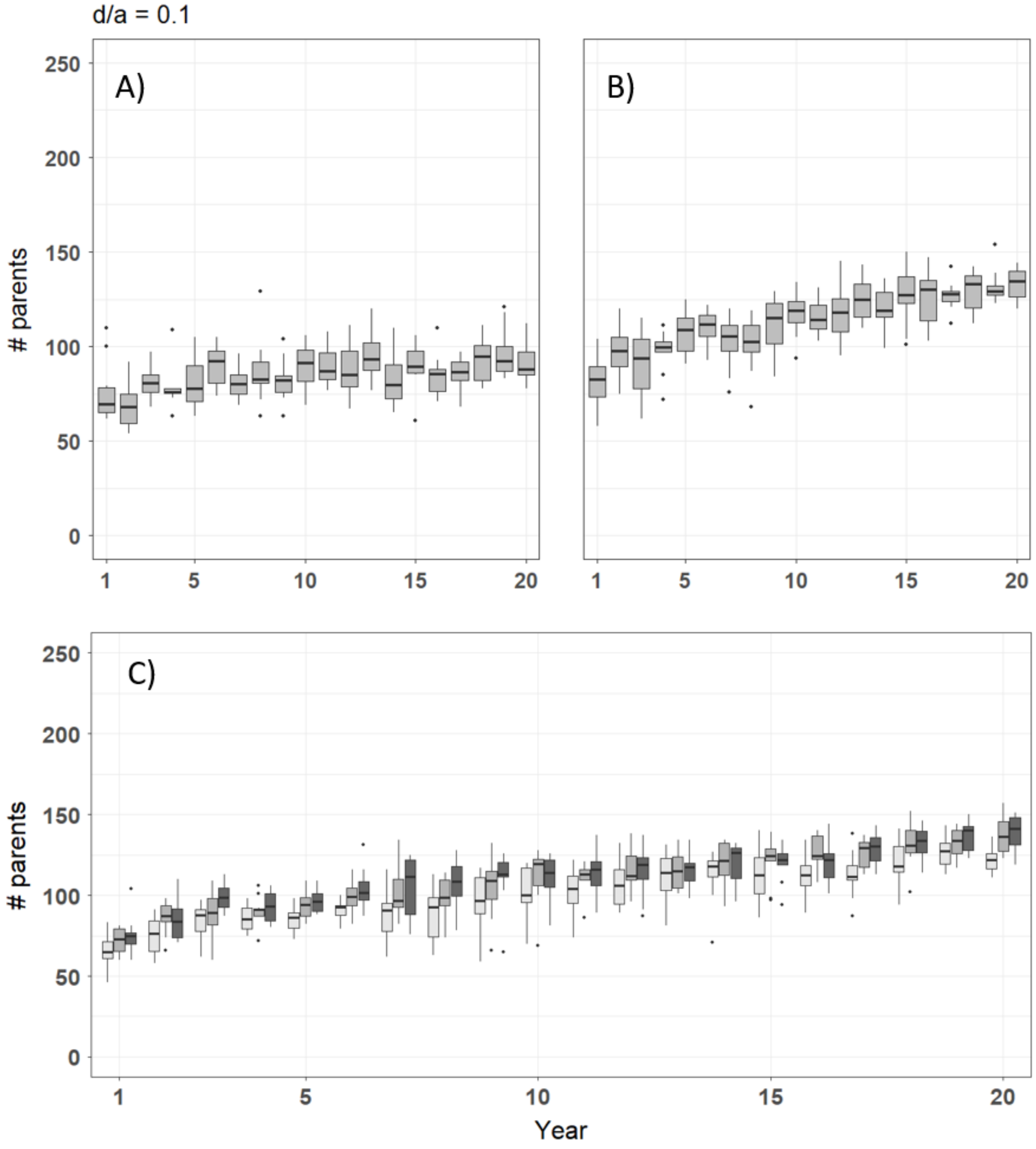

Figure S5 Number of parents used in the breeding programs with parent selection based on genomic predicted cross performance (GPCP) under a dominance degree of 0.1. The number of parents used in the 10 simulation replications is shown as boxplots for the conventional breeding program with genomic selection (A), the two-part breeding program with one crossing cycle per year (B), and the two-part breeding program with three crossing cycles per year (C) during the future breeding phase.

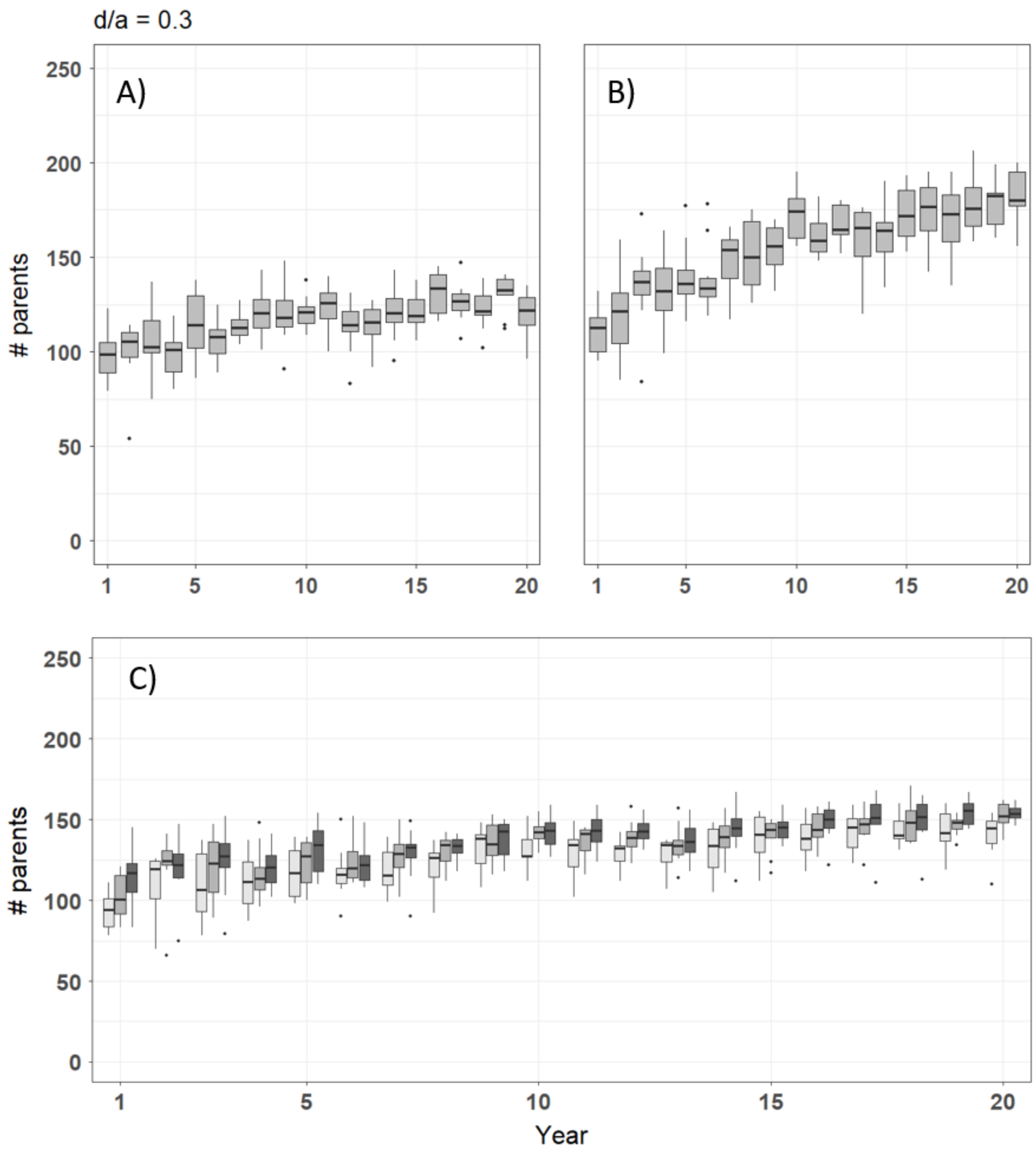

Figure S6 Number of parents used in the breeding programs with parent selection based on genomic predicted cross performance (GPCP) under a dominance degree of 0.3. The number of parents used in the 10 simulation replications is shown as boxplots for the conventional breeding program with genomic selection (A), the two-part breeding program with one crossing cycle per year (B), and the two-part breeding program with three crossing cycles per year (C) during the future breeding phase.

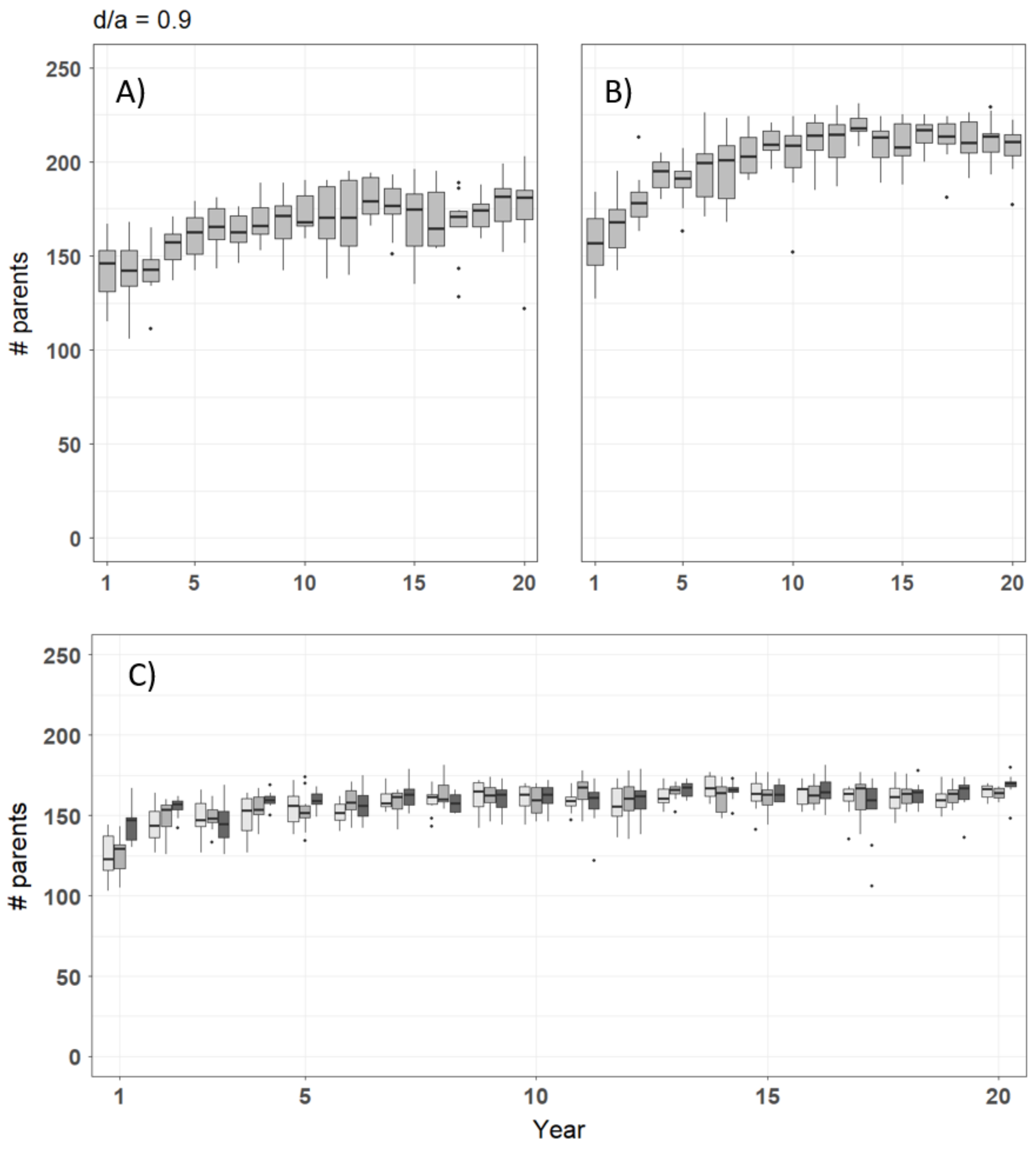

Figure S7 Number of parents used in the breeding programs with parent selection based on genomic predicted cross performance (GPCP) under a dominance degree of 0.9. The number of parents used in the 10 simulation replications is shown as boxplots for the conventional breeding program with genomic selection (A), the two-part breeding program with one crossing cycle per year (B) and the two-part breeding program with three crossing cycles per year (C) during the future breeding phase.
