# Supplementary material for: Genomic selection strategies for clonally propagated crops
Source: Theor Appl Genet. 2023 Mar 23;136(4):74. doi: 10.1007/s00122-023-04300-6 (PMC10036424; doi:10.1007/s00122-023-04300-6)
Supplement: Supplementary file 3 — Supplementary file3 (PDF 540 KB) [file 122_2023_4300_MOESM3_ESM.pdf]

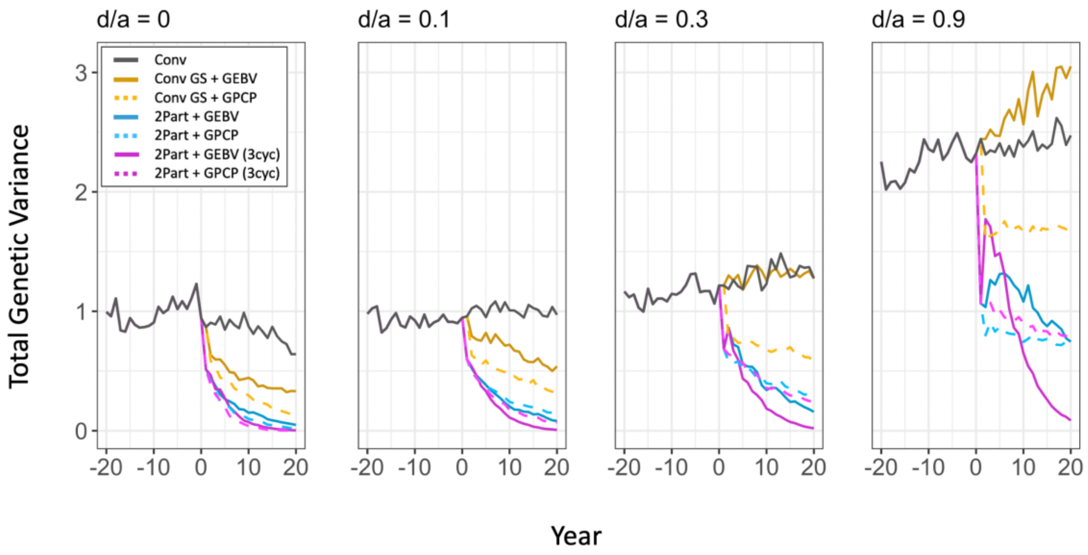

Figure S8 Total genetic variance of the simulated breeding programs under different dominance degrees (d/a). In each panel, total genetic variance is plotted in stage 1 for the entire burn-in breeding phase and the future breeding phase. Each line shows the mean genetic variance for the 10 simulated replications. The different types of breeding program are shown in different colours. The conventional breeding program (Conv) is gray. The conventional breeding program with genomic selection (Conv GS) is yellow. The two-part breeding program with genomic selection (2Part) is shown in blue with one crossing cycle per year and in purple with three crossing cycles per year. The two types of parent selection were shown in different line-styles. Selection based on genomic estimated breeding values (GEBV) is shown by continuous lines. Selection based on genomic prediction of cross performance (GPCP) is shown by dashed lines.

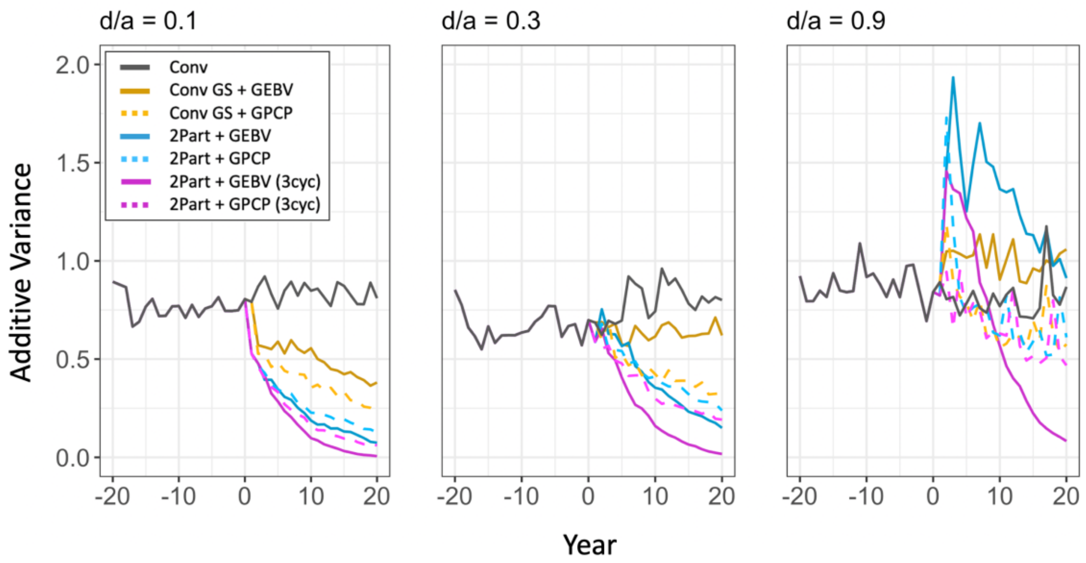

Figure S9 Additive variance of the simulated breeding programs under different dominance degrees (d/a). In each panel, additive variance is plotted in stage 1 for the entire burn-in breeding phase and the future breeding phase. Each line shows the mean additive variance for the 10 simulated replications. The different types of breeding program are shown in different colours. The conventional breeding program (Conv) is gray. The conventional breeding program with genomic selection (Conv GS) is yellow. The two-part breeding program with genomic selection (2Part) is shown in blue with one crossing cycle per year and in purple with three crossing cycles per year. The two types of parent selection were shown in different line-styles. Selection based on genomic estimated breeding values (GEBV) is shown by continuous lines. Selection based on genomic prediction of cross performance (GPCP) is shown by dashed lines.

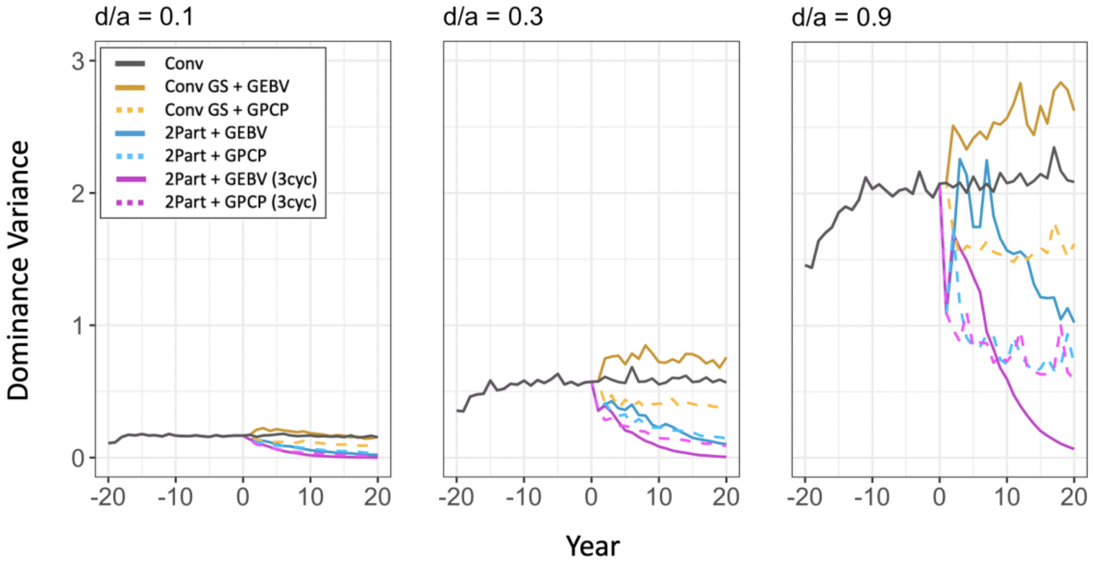

Figure S10 Dominance variance of the simulated breeding programs under different dominance degrees (d/a). In each panel, dominance variance is plotted in stage 1 for the entire burn-in breeding phase and the future breeding phase. Each line shows the mean dominance variance for the 10 simulated replications. The different types of breeding program are shown in different colours. The conventional breeding program (Conv) is gray. The conventional breeding program with genomic selection (Conv GS) is yellow. The two-part breeding program with genomic selection (2Part) is shown in blue with one crossing cycle per year and in purple with three crossing cycles per year. The two types of parent selection were shown in different line-styles. Selection based on genomic estimated breeding values (GEBV) is shown by continuous lines. Selection based on genomic prediction of cross performance (GPCP) is shown by dashed lines.
