# Supplementary material for: Genomic selection strategies for clonally propagated crops
Source: Theor Appl Genet. 2023 Mar 23;136(4):74. doi: 10.1007/s00122-023-04300-6 (PMC10036424; doi:10.1007/s00122-023-04300-6)
Supplement: Supplementary file 4 — Supplementary file4 (PDF 107 KB) [file 122_2023_4300_MOESM4_ESM.pdf]

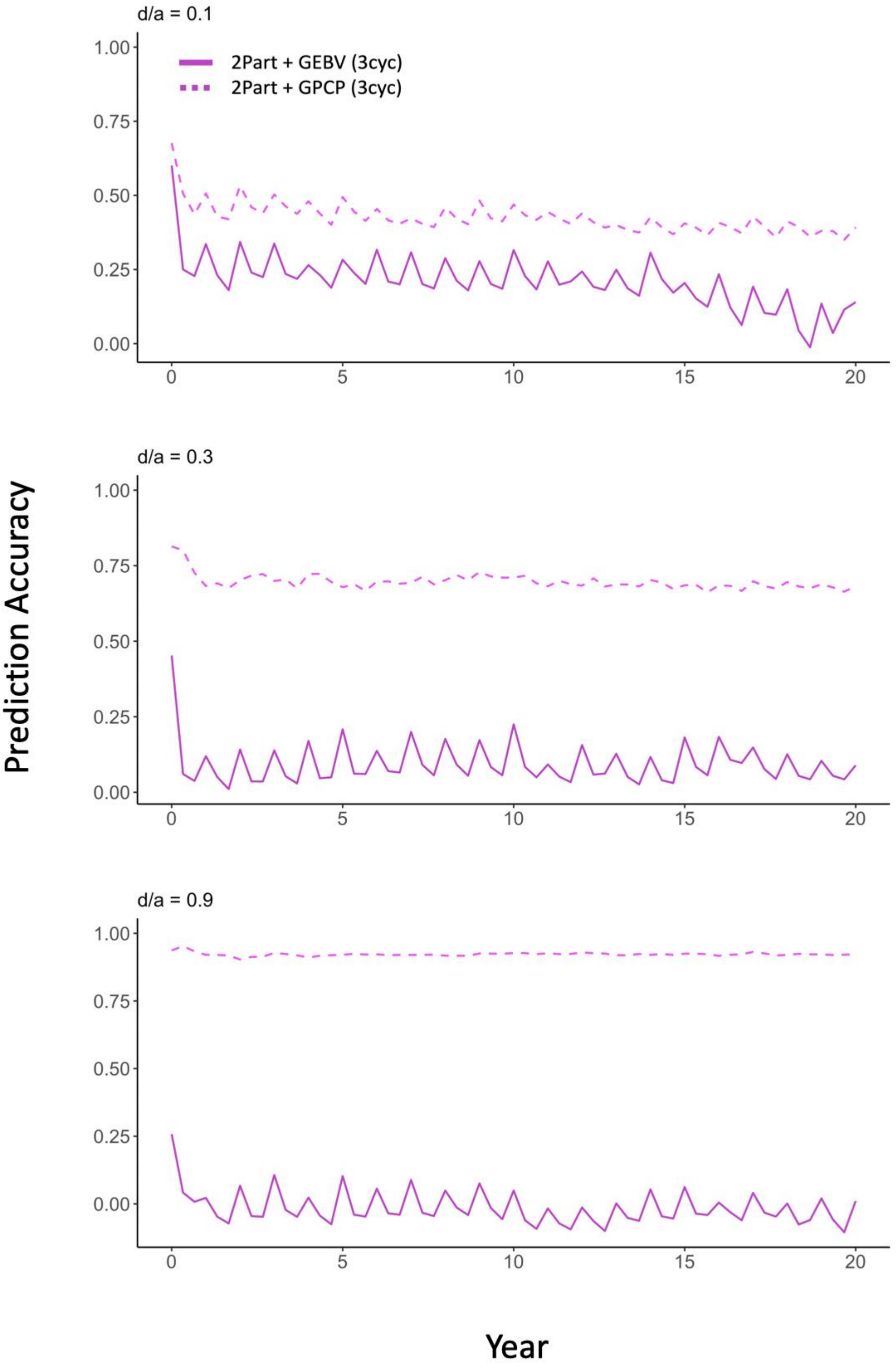

Figure S11 Prediction accuracy for selection of new parents in the two-part breeding programs with three crossing cycles per year under different dominance degrees (d/a). In each panel, prediction accuracy is plotted for the future breeding phase of the two-part breeding programs with three crossing cycles per year. Each line shows the mean prediction accuracy for the 10 simulated replications of the two

breeding programs at all three crossing cycles per year. The two types of parent selection were shown in different line-styles. Selection based on genomic estimated breeding values (GEBV) is shown by continuous lines. Selection based on genomic prediction of cross performance (GPCP) is shown by dashed lines. Prediction accuracy was measured in the seedling stage.
