# Supplementary material for: Genomic selection strategies for clonally propagated crops
Source: Theor Appl Genet. 2023 Mar 23;136(4):74. doi: 10.1007/s00122-023-04300-6 (PMC10036424; doi:10.1007/s00122-023-04300-6)
Supplement: Supplementary file 5 — Supplementary file5 (PDF 10 KB) [file 122_2023_4300_MOESM5_ESM.pdf]

Table S2 Assessment of the prediction accuracy of the parent selection method in the breeding programs with genomic selection. Prediction accuracy was measured as Pearson correlation between the parent selection method and the true value.

Breeding program	Parent selection stage	Parent selection method	True value
**Conv GS**	Clonal stage 1	GEBV of one parent	Breeding value
	Clonal stage 1	GPCP of cross between two parents	Mean genetic value of the F1
**2Part**	Seedling stage	GEBV of one parent	Breeding value
	Seedling stage	GPCP of cross between two parents	Mean genetic value of the F1

Conv GS, conventional breeding program with genomic selection; 2Part, two-part breeding program, GEBV, genomic estimated breeding value; GPCP, genomic predicted cross performance.
