# Supplementary material for: Genomic selection strategies for clonally propagated crops
Source: Theor Appl Genet. 2023 Mar 23;136(4):74. doi: 10.1007/s00122-023-04300-6 (PMC10036424; doi:10.1007/s00122-023-04300-6)
Supplement: Supplementary file 6 — Supplementary file6 (PDF 29 KB) [file 122_2023_4300_MOESM6_ESM.pdf]

Table S3 Assessment of the prediction accuracy of the total genetic value in the seedling stage for advancement of seedlings to clonal stage 1. Prediction accuracy was measured as Pearson correlation between the seedling selection method applied in the seedling stage and the true total genetic value.

Breeding Program	Seedling selection method	True value
**Conv**	Phenotype	Total genetic value
**Conv GS**	Phenotype	Total genetic value
**2Part**	GEGV	Total genetic value

Conv, conventional breeding program; Conv GS, conventional breeding program with genomic selection; 2Part, two-part breeding program, GEGV, genomic estimated genetic value.
